# Inflammasome Inhibitor MCC950 Attenuates Methamphetamine-Induced Hippocampal Neurotoxicity and Aberrant Neurogenesis in a Sex-Dependent Manner

**DOI:** 10.3390/cells15161443

**Published:** 2026-08-11

**Authors:** Mateusz Smolarz, Natalia Pondel, Gracjana Zając, Agata Kurczyk, Monika Pietrowska, Marta Gawin, Magdalena Dębiec, Andrzej Małecki, Marta Nowacka-Chmielewska, Michal Toborek

**Affiliations:** 1 Institute of Physiotherapy and Health Sciences, Academy of Physical Education, 40-065 Katowice, Poland; m.smolarz@awf.katowice.pl (M.S.); n.pondel@awf.katowice.pl (N.P.); gracjana.zajac@gmail.com (G.Z.); d201110@365.sum.edu.pl (M.D.); a.malecki@awf.katowice.pl (A.M.); 2 Maria Sklodowska-Curie National Research Institute of Oncology, Gliwice Branch, 44-102 Gliwice, Poland; m.pietrowska@igbzpan.pl (M.P.); m.gawin@igbzpan.pl (M.G.); 3 Department of Biostatistics and Bioinformatics, Maria Skłodowska-Curie National Research Institute of Oncology, Gliwice Branch, 44-102 Gliwice, Poland; agata.kurczyk@gliwice.nio.gov.pl; 4 Department of Biochemistry and Molecular Biology, University of Miami Miller School of Medicine, Miami, FL 33136, USA

**Keywords:** inflammasome, neuroinflammation, MCC950, methamphetamine, behavior, cognition

## Abstract

**Highlights:**

**What are the main findings?**

- MCC950 produced beneficial outcomes at both the behavioral and inflammatory levels, suppressing inflammatory activation and enhancing hippocampal cell proliferation.
- The effects of MCC950 were more pronounced in male mice, indicating sexual dimorphism.

**What are the implications of the main findings?**

- This study underscores the potential of targeting inflammasome activation as a preventive strategy against METH-induced neurotoxicity and highlights the importance of considering sex differences when developing inflammasome-targeted therapeutic approaches.

**Abstract:**

Methamphetamine (METH) is a known proinflammatory agent; however, the impacts of inflammasomes on its neurotoxic effects are not fully understood. In the present study, we assessed the impact of prolonged METH administration on the hippocampal inflammasome profile in male and female mice and determined alterations of the inflammasome profile in response to METH. In addition to inflammasome activation, METH induced both systemic and hippocampal-specific inflammatory responses, leading to cognitive impairment, reduced hippocampal cell proliferation, and altered proteomic profiles. Importantly, the responses to METH exposure exhibited important sexual dimorphism. Treatment with inflammasome inhibitor MCC950 attenuated METH-induced inflammatory events; however, we also observed several off-target effects of this inhibitor affecting mouse anxiety-like behavior and cognitive function. Overall, our results indicate the preventive potential of MCC950 in METH-related neurotoxicity, while underscoring its limitations due to distinct sex-dependent differences in response to both METH and MCC950 and highlighting significant sexual dimorphism.

## 1. Introduction

Methamphetamine (METH), one of the most common psychostimulants worldwide, is characterized by high neurotoxicity, which is, at least in part, related to the excessive release of serotonin [1] and dopamine [2]. The dysfunction of the ubiquitin–proteasome system, increased protein nitration and reticular stress, and loss of blood–brain barrier (BBB) integrity were reported in an animal model [3] and primary human brain endothelial cell culture in response to METH treatment [4,5]. Increasing evidence suggests that METH-induced inflammatory responses can be a primary factor orchestrating these effects [6]. Moreover, the METH-induced overproduction of inflammatory cytokines, especially IL-1β [7,8], has been linked to cognitive decline.

Inflammasomes are cytoplasmic protein complexes involved in innate immunity; however, their prolonged overactivation during neuroinflammation can drive pathological inflammation and cell death and contribute to the pathological processes of various neurodegenerative diseases [9,10,11,12]. Inflammasomes, such as AIM2, NLRC4, NLRP1, and NLRP3, have been identified to play key roles in neuroinflammatory processes in the central nervous system [13,14]. Inflammasomes are activated by diverse triggers, ranging from infectious pathogens and bacterial toxins to environmental stressors [12]. The components of inflammasomes are widely expressed in microglia [15], astrocytes, and neurons [16]. Inflammasome activation involves a two-step process, beginning with NF-κB signaling, which leads to transcription and post-translational priming [17]. Activated inflammasomes promote caspase-1 maturation [18,19], which processes pro-IL-1β and pro-IL-18 into their active forms [20].

An impaired hippocampal morphology and function have been associated with cognitive deficits observed after chronic METH use and seem to play a critical role in increasing the propensity for relapse [7,21,22,23]. In addition, METH can affect hippocampal neurogenesis by interfering with the life cycle and survival of neural stem cells (NSCs) [24,25]. The role of neurogenesis in the dentate gyrus in promoting the reinstatement of METH-seeking behavior has also been emphasized [26]. Finally, elevated NLRP1 and NLRP3 levels and the induction of neuronal apoptosis have been found in the hippocampi of animals exposed chronically to METH [25,26].

The inhibition of NLRP3 inflammasome activation with MCC950 has been proposed as an effective strategy for the treatment of inflammatory responses [27]. Because of the strong inflammatory component of METH-induced toxicity, we employed a similar treatment strategy in the present study. Importantly, MCC950 is BBB-permeable and has been successfully used to treat several CNS disorders. Preclinical studies have shown the therapeutic effects of MCC950 in models of cardiovascular diseases [28,29], inflammatory diseases [30,31], autoimmune diseases [32], and perioperative neurocognitive disorders [33]. In addition, a single study has proposed that the inhibition of NLRP3 inflammasome activation can prevent motor deficits and cerebellar pathologies induced by the chronic administration of METH [34].

In the present study, we identified that inflammasome inhibition with MCC950 attenuated METH-induced neuroinflammation, aberrant neurogenesis, and cognitive decline. Importantly, our results demonstrated prominent sexual dimorphism in the METH-induced upregulation of inflammatory responses and cognitive impairment.

## 2. Materials and Methods

### 2.1. Animals

Male and female nine-week-old C57BL/6 mice (Mus musculus) were provided by the Animal House of the Department for Experimental Medicine, Medical University of Silesia, Katowice, Poland. Mice underwent one week of habituation before the start of the experiments. All experimental procedures received approval from the Local Committee for the Care and Use of Laboratory Animals (permission no. 51/2021, 23 September 2021, Katowice, Poland). Animals were housed 3–4 per cage in a climate-controlled room under standard conditions (temp.: 22 ± 2 °C, relative humidity: 55 ± 10%, 12 h day/night cycle with light presented at 7:00 a.m.). The experiments took place during the light cycle, and animals had free access to water and standard chow.

### 2.2. Experimental Design

**Figure 1:**
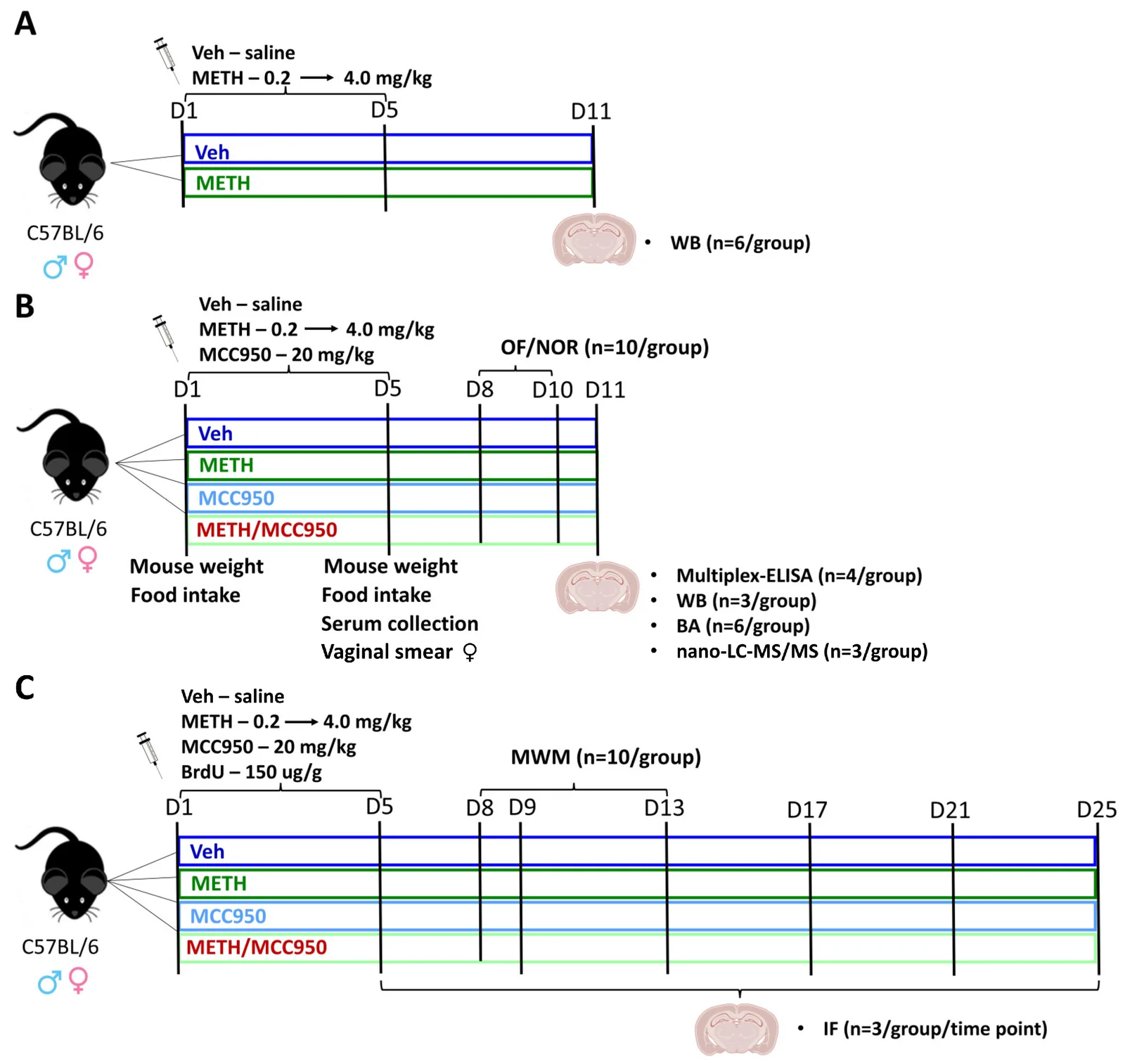
Experimental design and timeline of the study. (**A**) In the first set of experiments, we determined the inflammasome profile in the hippocampi of mice exposed to METH. (**B**) The second set of experiments evaluated the impacts of MCC950 administration on METH-induced inflammatory responses, BBB-related tight junction proteins, neuronal differentiation, the hippocampal proteome, and cognitive abilities. (**C**) The third set of experiments assessed the effects of MCC950 administration on hippocampal cell proliferation and the immunoreactivity of NG2-positive cells, as well as spatial memory. Abbreviations: WB, Western blot; BA, biochemical analysis (AST and ALT); Multiplex-ELISA, multiplex cytokine bead-based enzyme-linked immunosorbent assay; Nano-LC-MS/MS, nanoscale liquid chromatography–tandem mass spectrometry; IF, immunofluorescence; OF, open field; NOR, novel object recognition; MWM, Morris water maze.

The experimental design of the study is presented in Figure 1. A total of 248 animals (124 males and 124 females) were used in the experiments. In the first set of experiments (Figure 1A), mice were injected intraperitoneally (i.p.) with a vehicle (Veh, saline solution) or METH for 5 consecutive days, creating the Veh and METH groups, respectively. Then, the animals were euthanized on day 11 (D11), and hippocampal tissues were collected.

In the second set of experiments (Figure 1B), we evaluated the impacts of an inflammasome inhibitor, MCC950, on METH-induced inflammatory responses, BBB-related tight junction proteins, neuronal differentiation, the hippocampal proteome, and behavior. On day 1 (D1) of the study, the body weights of the mice and food consumption were recorded. The animals were divided into the groups Veh, METH, MCC950, and METH/MCC950, which received daily i.p. injections with the vehicle, METH, vehicle + MCC950, or METH + MCC950, respectively, from D1 to D5. On D5, the reassessment of animal body mass and food intake was performed, and both vaginal smears and tail vein blood samples were collected. The mice underwent open field (OFT) and novel object recognition (NOR) testing between D8 and D10. The animals were sacrificed on day 11 and hippocampal tissues were collected.

In the final set of experiments (Figure 1C), additional injections with BrdU were administered once a day for 5 days during the METH exposure period to assess the impacts of MCC950 on cell proliferation and the immunoreactivity of hippocampal NG2-positive cells. Mice were euthanized every 4 days after the end of the injections, i.e., they were euthanized on days 5, 9, 13, 17, 21, and 25. Their brains were collected for further immunofluorescence studies. The same cohort of animals also underwent the Morris water maze (MWM) test from D8 to D13.

### 2.3. Administration of METH, MCC950, and BrdU

All substances were administered i.p. METH (cat # M8750, Sigma-Aldrich, St. Louis, MA, USA) was injected to the mice three times over four consecutive days, utilizing an escalating dosing regimen that increased by 0.2 mg/kg per injection, from an initial 0.2 mg/kg to a final 2.4 mg/kg dose. On D5, mice were injected three times with a high dose of METH (4.0 mg/kg) at 4 h intervals. The stepwise increase in METH intake reflects the pattern of human drug use and has been previously described in the literature [7,21,35,36]. An equivalent volume of saline solution was injected in the control group (cat # 100038629, Fresenius Kabi, Warsaw, Poland). Inflammasome inhibitor MCC950 (20 mg/kg, cat # M13769-200, Molnova, Anna Arbor, MI, USA) was injected once a day for five days during the administration of METH or the vehicle. For the experiments assessing cell proliferation, injections of BrdU (cat # 10280879001, Roche, Mannheim, Germany) at 150 μg/g, once a day for 5 days, were performed (Figure 1).

### 2.4. Vaginal Smears

The phases of the estrous cycle were determined by the vaginal smears collected on D5, as described previously [36]. Similar handling was introduced in male mice to minimize potential differences in cross-sex data interpretation. The vaginal smears (*n* = 10 per group) were examined with the EVOS™ XL Core Imaging System at 20× magnification (Thermo Fisher Scientific, Waltham, MA, USA). As presented in Supplementary Table S1, 47.4% of female mice subjected to the OFT and NOR tests were in the diestrus phase, 36.8% were in the metestrus phase, 15.8% were in the estrus phase, and none of the mice were in the proestrus phase. In addition, 24.4% of mice subjected to the MWM were in the diestrus phase, 65.9% in the metestrus phase, 7.3% in the estrus phase, and 2.4% in the proestrus phase.

### 2.5. Serum and Tissue Collection

On D5, tail vein blood was collected in Microvette^®^ 200 tubes with separating gel (cat # 20.1291, Sarstedt, Germany) and spun to obtain serum. Serum samples were divided into aliquots and stored at −80 °C until further analyses (Figure 1B).

For the experiments visualized in Figure 1A,B, the animals were euthanized under anesthesia via decapitation. Hippocampal tissues were isolated using a 1 mm Stainless Steel Brain Matrix (cat # 15067, Ted Pella, Inc., Redding, CA, USA) based on coordinates derived from the Mouse Brain in Stereotaxic Coordinates: Compact Second Edition (Elsevier, Amsterdam, The Netherlands), in the bregma range of −1.40 mm to −3.40 mm. Protein lysates from hippocampal samples were prepared as described earlier [36]. Protein levels were assessed with Pierce BCA Protein Assay Kits (cat # 23227, Thermo Fisher Scientific, Waltham, MA, USA). The obtained lysates were subsequently analyzed via Western blotting, nanoscale liquid chromatography–tandem mass spectrometry, and Multiplex-ELISA.

For the experiments reflected in Figure 1C, mice underwent anesthesia with ketamine (100 mg/kg) and xylazine (10 mg/kg) and were then perfused through the left ventricle with ice-cold PBS followed by 4% paraformaldehyde (PFA). The perfusion process was maintained until the entire body became completely rigid. Following extraction, brains were post-fixed in 4% PFA for 24 h and stored at 4 °C in 0.02% sodium azide until further analysis.

### 2.6. Western Blotting

An equal mass of protein lysate (10 µg) was used for immunoblotting, according to the protocol described previously [36]. Primary and secondary antibodies, including catalog numbers and dilutions, are listed in Supplementary Table S2. Protein bands were subsequently quantified using the Image Lab 6.0.1 software (Bio-Rad, Hercules, CA, USA) and normalized to the total protein content in the well.

### 2.7. Biochemical Analysis

AST and ALT enzymatic activity was assessed by the kinetic UV method using the Beckman Coulter AU680. (Brea, CA, USA).

### 2.8. Multiplex Cytokine Bead-Based Enzyme-Linked Immunosorbent Assay

Inflammatory cytokines were assessed in serum and hippocampal tissue using a mouse Bio-Plex assay (cat # M60009RDPD, Bio-Rad, Hercules, CA, USA), in accordance with the manufacturer’s instructions.

### 2.9. Proteomic Profiling

#### 2.9.1. Preparation of Tissue Lysates for Nano-LC-MS/MS Analysis

Aliquots containing 50 µg of protein were prepared as described earlier [37]. Incubation with the enzyme mix was conducted for 18 h at 37 °C. The obtained peptides were subsequently pre-concentrated using C18 StageTips [38], each containing 8 pieces of Empore SPE Disk matrix active group C18. Peptides eluted from the bed using a mixture of 60% ACN and 0.1% TFA were then dried in a vacuum concentrator, reconstituted in 40 µL of pure water, and subjected to peptide assay using the tryptophan fluorescence method [39]; they were then acidified with trifluoroacetic acid (TFA, 0.1%). Before nano-LC-MS/MS measurements, all peptide samples were diluted with 0.1% TFA to obtain 1 µg of peptide per injection for each sample.

#### 2.9.2. Nano-LC-MS/MS Measurements and Proteomic Data Preprocessing

A Q-Exactive Plus mass spectrometer coupled with an RSLC UltiMate 3000 nano-liquid chromatograph and a Nanospray Flex ion source (all from Thermo Scientific) was used for measurements. Peptides from each fraction were separated on a reverse-phase Acclaim PepMap RSLC nanoViper C18 column (Thermo Fisher Scientific, Waltham, MA, USA) (75 μm × 50 cm, 2 μm granulation) using an acetonitrile gradient (from 8 to 35%, in 0.1% formic acid for 210 min) at 35 °C and a flow rate of 300 nL/min (total run time: 240 min). The spectrometer was operated in data-dependent MS/MS mode with survey scans acquired at a resolution of 70,000 at *m*/*z* 200 in MS mode and 17,500 at *m*/*z* 200 in MS2 mode. Spectra were recorded in the scanning range of 300–2000 *m*/*z* in positive ion mode. Higher-energy collisional dissociation (HCD) ion fragmentation was performed, with the normalized collision energies set to 25. Protein identification was performed using a reviewed Swiss-Prot mouse database (release 2021_05_14, containing 17,050 sequence entries and 9,670,359 residues) with a precision tolerance of 10 ppm for peptide masses and 0.02 Da for fragment ion masses. All raw data obtained for each dataset were imported into Proteome Discoverer v.1.4 (Thermo Fisher Scientific) “Thermo raw files” for protein identification and quantification (the Sequest engine was used for database searches). A protein was considered positively identified if at least two peptides per protein were found and the peptide score reached the significance threshold FDR = 0.01 (assessed by the Percolator algorithm); a protein was further considered “present” if detected in at least one sample of a given type. The abundances of identified proteins were estimated in Proteome Discoverer using the Precursor Ions Area detector node, which calculates the abundance of a given protein based on the average intensity of the three most intensive distinct peptides for this protein, with further normalization to the total ion current (TIC). The high-resolution mass spectrometry-based proteomic data have been deposited with the ProteomeXchange Consortium via the PRIDE (https://www.ebi.ac.uk/pride, accessed on 25 July 2025) partner repository with the dataset identifier PXD066569 [40,41].

### 2.10. Proteomics Data Analysis

#### 2.10.1. Data Preprocessing

Measurements below the detection limit were treated as missing values and imputed using random numbers drawn from a lognormal distribution. The distribution parameters were estimated using the maximum likelihood method, considering the left truncation between 0 and the lowest non-missing measured value for each protein. Proteins with more than one missing value per group (i.e., >33% missing data in any group) were excluded from quantitative analysis and analyzed using binary methods instead.

#### 2.10.2. Statistical Hypothesis Testing

Protein abundances were analyzed separately for male and female mice using a robust two-way ANOVA on medians with factors METH and MCC950, which provided resistance to violations of normality and heteroscedasticity (2494 proteins for males and 2516 proteins for females, each with ≤33% missing values per group). For proteins exhibiting a significant interaction, four predefined post hoc comparisons were performed: the effects of METH in the absence and presence of MCC950 and the effects of MCC950 in the absence and presence of METH. Post hoc comparisons were conducted using robust, bootstrap-based tests on medians. Effect sizes were calculated using Cliff’s delta for pairwise comparisons to assess simple effects, with interpretation based on the following thresholds: negligible effect (|δ| < 0.15), small effect (|δ| ≥ 0.15), medium effect (|δ| ≥ 0.33), and large effect (|δ| ≥ 0.47). The Jonckheere–Terpstra test [42] was used to assess monotonic trends across an ordinal group variable. A priori biological ordering of the four experimental groups (MCC950 < Veh < METH/MCC950 < METH) was applied based on the expected progression of METH-induced neuroinflammation, with the lowest inflammatory activation in MCC950-treated mice and the highest in METH-treated mice. This approach allowed us to specifically identify proteins whose expression exhibited a directional trend consistent with the hypothesized increase in inflammatory activation.

Fisher’s exact test was applied to evaluate whether the absence/presence status of the remaining 567 and 544 proteins for males and females, respectively, was significantly associated with the experimental treatment groups. Cramér’s V was computed as a measure of the effect size for the Fisher’s exact test, interpreted according to Cohen’s thresholds [43] for three degrees of freedom: negligible (|r| < 0.06), small (|r| ≥ 0.06), medium (|r| ≥ 0.17), and large (|r| ≥ 0.29). The Cochran–Armitage test for trends [44] was used to examine whether the absence/presence status of a given protein was associated with the ordinal group variable (Veh, METH, MCC950, or METH/MCC950).

Two-sided statistical hypotheses were tested at a significance level of 5%. Multiple testing correction was performed using the Benjamini–Hochberg procedure, with adjusted *p*-values < 0.05 considered significant. All analyses were conducted using the R environment for statistical computing (version 4.4.1) and MATLAB software (version 2024a).

### 2.11. Immunofluorescence

The brains, previously kept in 0.02% sodium azide, were dehydrated through a series of sucrose solutions (10%, 20%, and 30%). Prepared brain slices were evaluated using immunofluorescence, as described previously [36]. The primary and secondary antibodies with which the slices were incubated are listed in Supplementary Table S2. Imaging was performed at 20× magnification using the ELYRA 7 system (Zeiss, Jena, Germany). The mean fluorescence within the outlined hippocampal areas was quantified automatically via the Zen 3.10 software. BrdU-positive cells were manually counted and expressed as the number of positive cells per μm^2^.

### 2.12. Behavioral Tests

Behavioral tests included the open field test (OFT), the novel object recognition (NOR) test, and the Morris water maze (MWM). All tests were performed 3 days after the last injection of METH, MCC950, or saline (Figure 1B,C). These tests were conducted under dim lighting (~10 lx) and suppressed noise conditions. The Ethovision XT 16.1 video tracking software (Noldus Information Technology, Wageningen, The Netherlands) was used for data recording and analysis. All behavioral tests were performed with *n* = 10 per group.

#### 2.12.1. OFT

The OFT was used to examine anxiety-like behavior and locomotor and exploratory activities. On D8, the animals were placed in a new, unknown room in a non-transparent square cage (44 × 44 cm), where they spent 10 min. During the OFT, we evaluated (a) the time spent in the center of the open field, (b) rearing, (c) grooming, (d) the total distance moved, and (e) the mean velocity.

#### 2.12.2. NOR

To investigate the integrity of memory and attention in mice, we conducted the NOR test (D9–10). The assay was performed as described earlier [7,36]. We assessed the % time with novel object, which expresses novel object exploration as a percentage of the total time: (novel object/total) × 100%.

#### 2.12.3. MWM

The MWM was performed to investigate spatial memory and learning abilities, as described earlier [7,36]. Memory retention was assessed by the following parameters: (a) escape latency, (b) mean velocity, (c) time spent in the targeted quadrant.

### 2.13. Statistical Analysis

All data, except for the proteomic data, were analyzed using the GraphPad Prism 10 or Statistica 13 software. The normality of each dataset was assessed by the Shapiro–Wilk test; after confirming that the data followed a normal distribution, differences between samples were assessed using one-, two-, three-, or four-way ANOVA. Tukey’s post hoc tests were applied only following statistically significant highest-order interactions in the respective ANOVA models. To ensure the clarity of the graphs, only statistically significant differences among Veh vs. METH, Veh vs. MCC950, and METH vs. METH/MCC950 are shown. For all analyses, *p*-values < 0.05 were considered statistically significant. Results are presented as mean ± SEM (*n* = 3–10 mice per group).

## 3. Results

We monitored general physiological parameters during 5-day exposure to METH and MCC950, while serum ALT and AST levels were assessed on the day of euthanasia (D11). Both male and female mice in the METH and METH/MCC950 groups exhibited a decrease in body weight. Moreover, food intake was reduced in both sexes in the METH group compared to the Veh group. In female mice, reduced food consumption was also observed in the MCC950 group relative to Veh (Supplementary Figure S1). However, the ALT and AST activity levels were not altered and thus did not indicate hepatotoxic effects of METH or MCC950 (Supplementary Figure S2).

### 3.1. Exposure to METH Alters Hippocampal Inflammasome Profile

**Figure 2:**
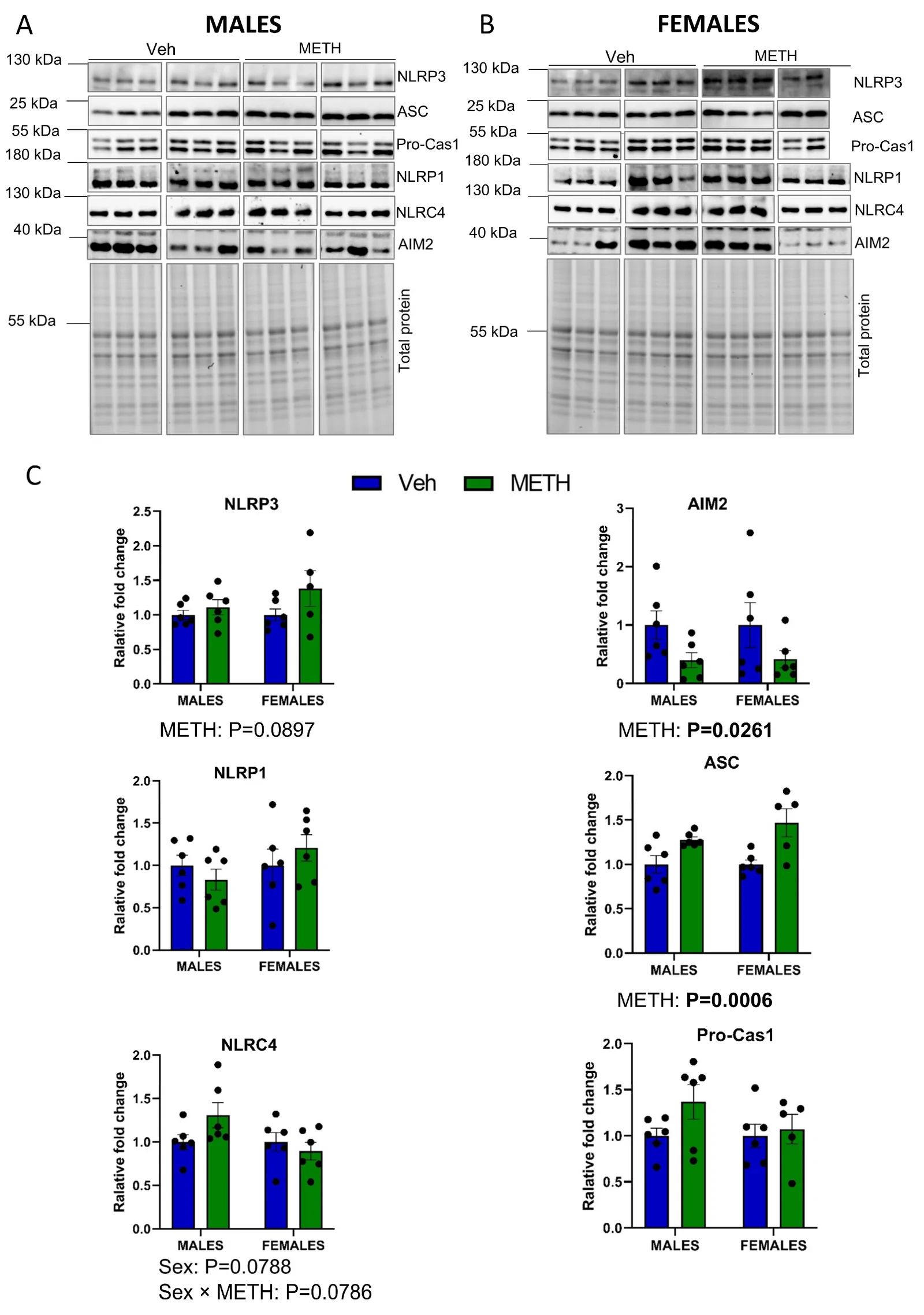
Impacts of METH administration on hippocampal expression of proteins forming inflammasome complexes. Mice were exposed to METH for 5 days and analyses were performed on the hippocampi collected on D11, as reflected in Figure 1A. Homogenates from dissected hippocampi were analyzed via immunoblotting to assess proteins forming inflammasome complexes (NLRP3, NLRP1, NLRC4, AIM2, ASC, and Pro-Cas1). Blots for male (**A**) and female (**B**) mice. (**C**) Quantitative immunoblotting data for males and females. Graphs represent the mean ± SEM. Statistical analysis was performed using a two-way ANOVA; *n* = 5–6 per each experimental group. Abbreviations: NLRP1 or NLRP3, NLR Family Pyrin Domain Containing 1 or 3; NLRC4, NLR Family CARD Domain Containing 4; Aim 2, Absent in Melanoma 2; ASC, apoptosis-associated speck-like protein containing a CARD; Pro-Cas1, pro-caspase-1; METH, methamphetamine.

In the first set of experiments, we determined the impact of METH on the hippocampal expression of the major components of the inflammasomes, such as NLRP3, NLRP1, NLRC4, ASC, and AIM2. METH treatment showed a strong trend (*p* = 0.0897) of increasing the NLRP3 protein levels in both male and female mice. The protein levels of ASC were statistically elevated (*p* = 0.0006) in both sexes (Figure 2A–C). In contrast, the AIM2 protein levels significantly decreased (*p* = 0.0261), and the NLRP1 and NLRC4 proteins were not altered following METH treatment. The levels of pro-caspase 1 exhibited a strong tendency to be increased, especially in male mice. Detailed information about the two-way ANOVA tests performed is provided in Supplementary Table S3.

Due to the role of the NLRP3 inflammasome in neuropathology, we employed the inflammasome inhibitor MCC950 in the remaining series of experiments.

### 3.2. MCC950 Modulates Hippocampal Tight Junction Protein Expression in a Sex-Dependent Manner

**Figure 3:**
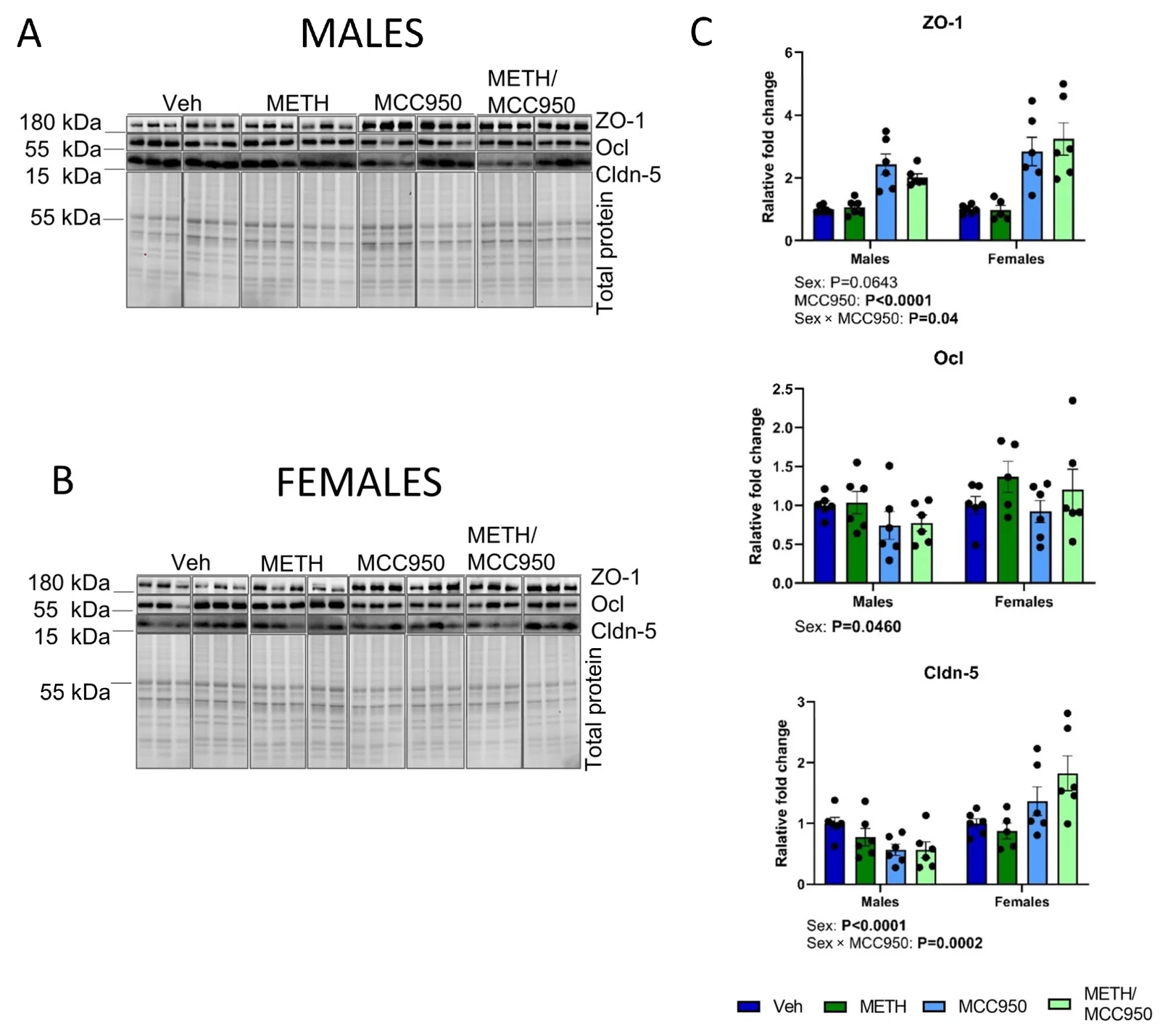
Impacts of METH and/or MCC950 administration on the hippocampal expression of BBB-related tight junction proteins. Mice were exposed to METH and/or MCC950 for 5 days, and analyses were performed on the hippocampi collected on D11, as reflected in Figure 1B. Homogenates from dissected hippocampi were analyzed by immunoblotting to assess the expression of tight junction proteins zona occludens-1 (ZO-1), occludin (Ocl), and claudin-5 (Cldn-5). Blots for male (**A**) and female (**B**) mice. Quantitative immunoblotting data for male and female mice (**C**). Graphs represent the mean ± SEM. Statistical analysis was performed using a three-way ANOVA; *n* = 5–6 per each experimental group.

To evaluate the impact of MCC950 treatment on BBB-related tight junction (TJ) proteins, we assessed the protein expression of zona ocludens-1 (ZO-1), occludin (Ocl), and claudin-5 (Cldn-5) in the hippocampus (Figure 3A–C). The results revealed that MCC950 affected TJ expression in a sex-dependent manner, as the interaction between sex and MCC950 was significant for Cldn-5 (*p* = 0.0002) and ZO-1 (*p* = 0.04). In addition, we identified a main effect of sex for Cldn-5 (*p* < 0.0001) and Ocl (*p* = 0.0460) and a tendency for ZO-1 (*p* = 0.0643). ZO-1 protein expression was profoundly affected by MCC950 (*p* < 0.0001). Detailed information about all ANOVA tests performed is provided in Supplementary Table S3.

### 3.3. Treatment with MCC950 Attenuates METH-Induced Systemic and Hippocampal Inflammatory Responses

**Figure 4:**
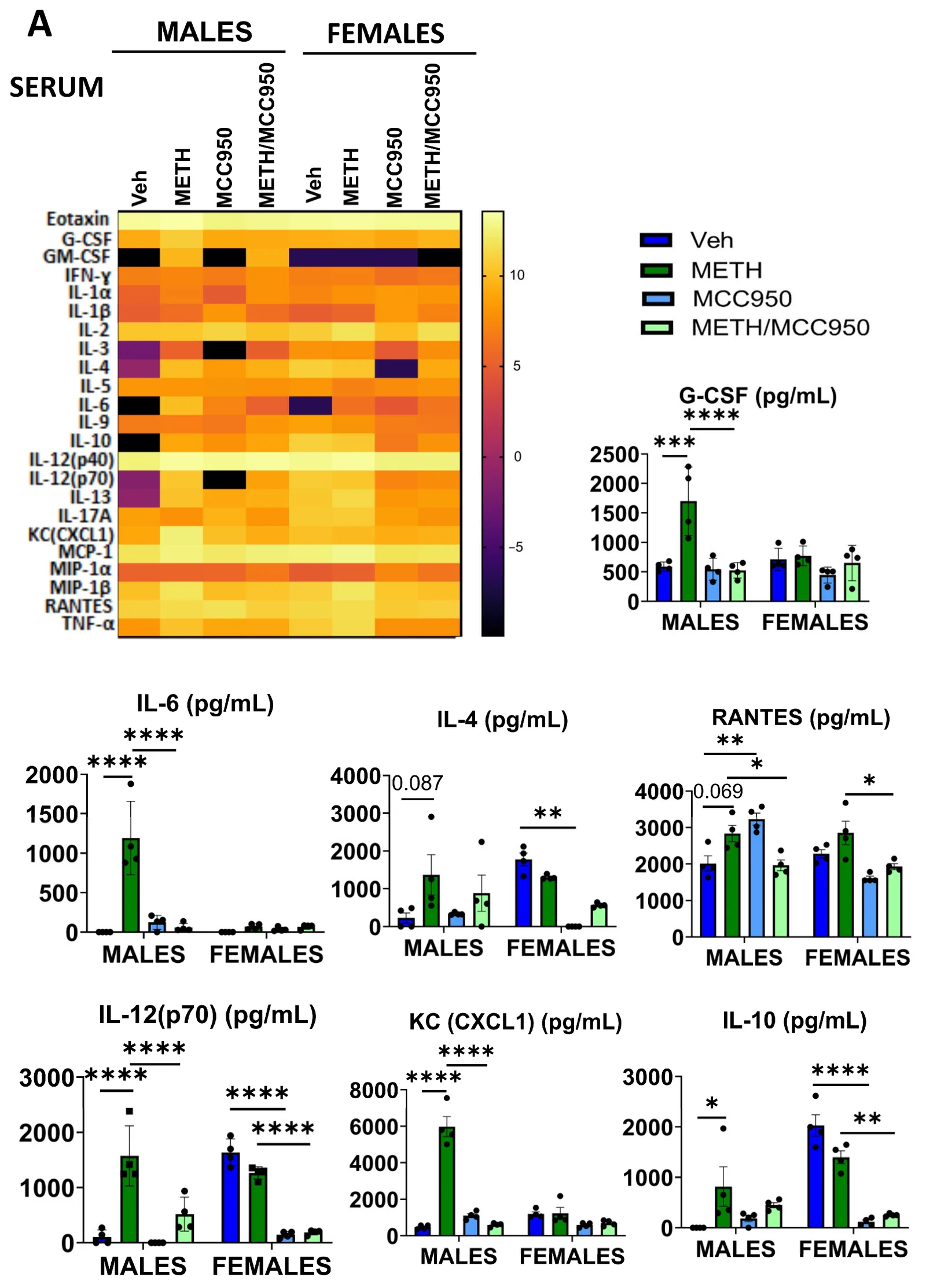

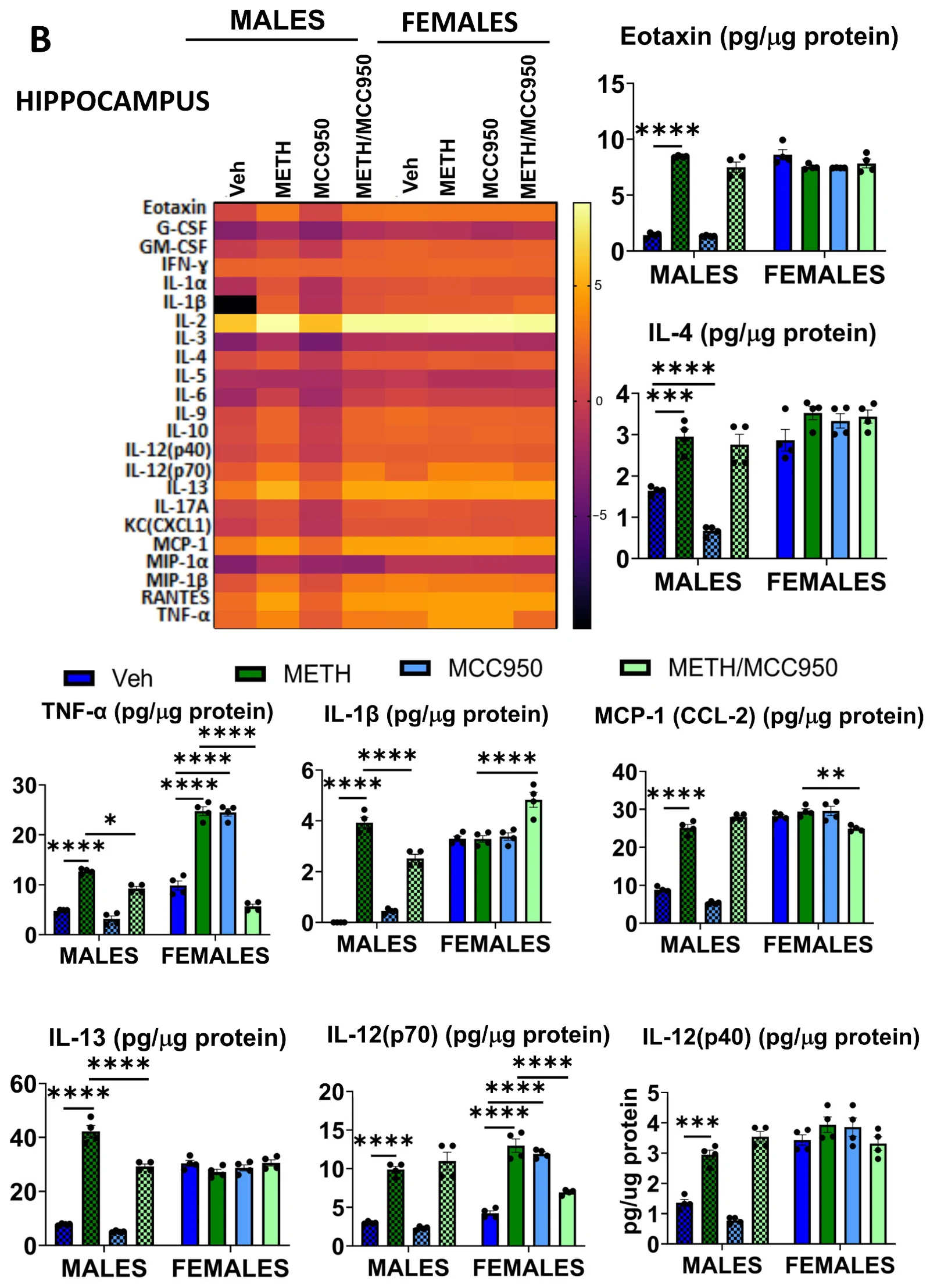
Impacts of MCC950 administration on METH-induced serum and hippocampal inflammatory cytokine levels. Mice were exposed to METH and/or MCC950 for 5 days as depicted in Figure 1B, and analyses were performed in mouse serum (**A**) and hippocampi (**B**). Heatmaps illustrate log2-transformed cytokine values, whereas bar graphs represent their quantitative levels. Data are means ± SEM. Statistical analysis was performed using three-way ANOVA followed by Tukey’s multiple-comparisons test; * *p* < 0.05, ** *p* < 0.01, *** *p* < 0.001, and **** *p* < 0.0001; *n* = 4 per experimental group. Abbreviations: IL, interleukin; G-CSF, granulocyte colony-stimulating factor; RANTES, regulated upon activation, normal T-cell expressed and secreted; KC, keratinocyte-derived chemokine; TNF, tumor necrosis factor; MCP-1, monocyte chemoattractant protein-1.

The impact of MCC950 on METH-induced inflammatory responses was evaluated using a panel of 23 proinflammatory cytokines in the serum and hippocampi of male and female mice (Figure 4). Detailed information about the performed ANOVA tests is provided in Supplementary Table S3.

The administration of METH and/or MCC950 resulted in distinct profiles of changes in the serum of female and male mice (Figure 4A). In male mice, METH administration resulted in the upregulation of the levels of G-CSF, GM-CSF, IL-6, IL-12(p70), IL-13, KC(CXCL1), MCP1(CCL2), MIP-1β, and TNF-α as compared to the vehicle group. In female mice, METH treatment significantly elevated only TNF-α levels compared to the control. Consistent with the heatmap analysis, the three-way ANOVA revealed significant METH × MCC950 × sex interactions for G-CSF, IL-4, IL-6, IL-10, IL-12(p70), KC (CXCL1), and RANTES. Importantly, the administration of MCC950 resulted in the attenuation of the METH-induced upregulation of cytokine levels or even lowering them below the control levels in both male and female mice.

In the hippocampi of male mice (Figure 4B), we observed the statistically significant upregulation of the levels of eotaxin, IL-1α, IL-1β, IL-2, IL-9, IL-10, IL-12(p40), IL-12(p70), IL-13, MCP1(CCL2), MIP-1β, RANTES, and TNF-α following METH administration as compared to the control. In contrast to serum, MCC950 treatment had a limited effect on the levels of these METH-induced cytokines. In the hippocampi of female mice, METH administration significantly elevated only the levels of IL-12(p70) and TNF-α. Interestingly, MCC950 treatment effectively protected against the upregulation of these cytokines in female mice. Importantly, eotaxin, IL-1β, IL-4, IL-12(p40), IL-12(p70) IL-13, MCP-1, and TNF-α exhibited a statistically significant METH x MCC950 x sex interaction in the three-way ANOVA.

### 3.4. Treatment with MCC950 Attenuates METH-Induced Aberrant Hippocampal Cell Proliferation and Neuronal Differentiation

**Figure 5:**
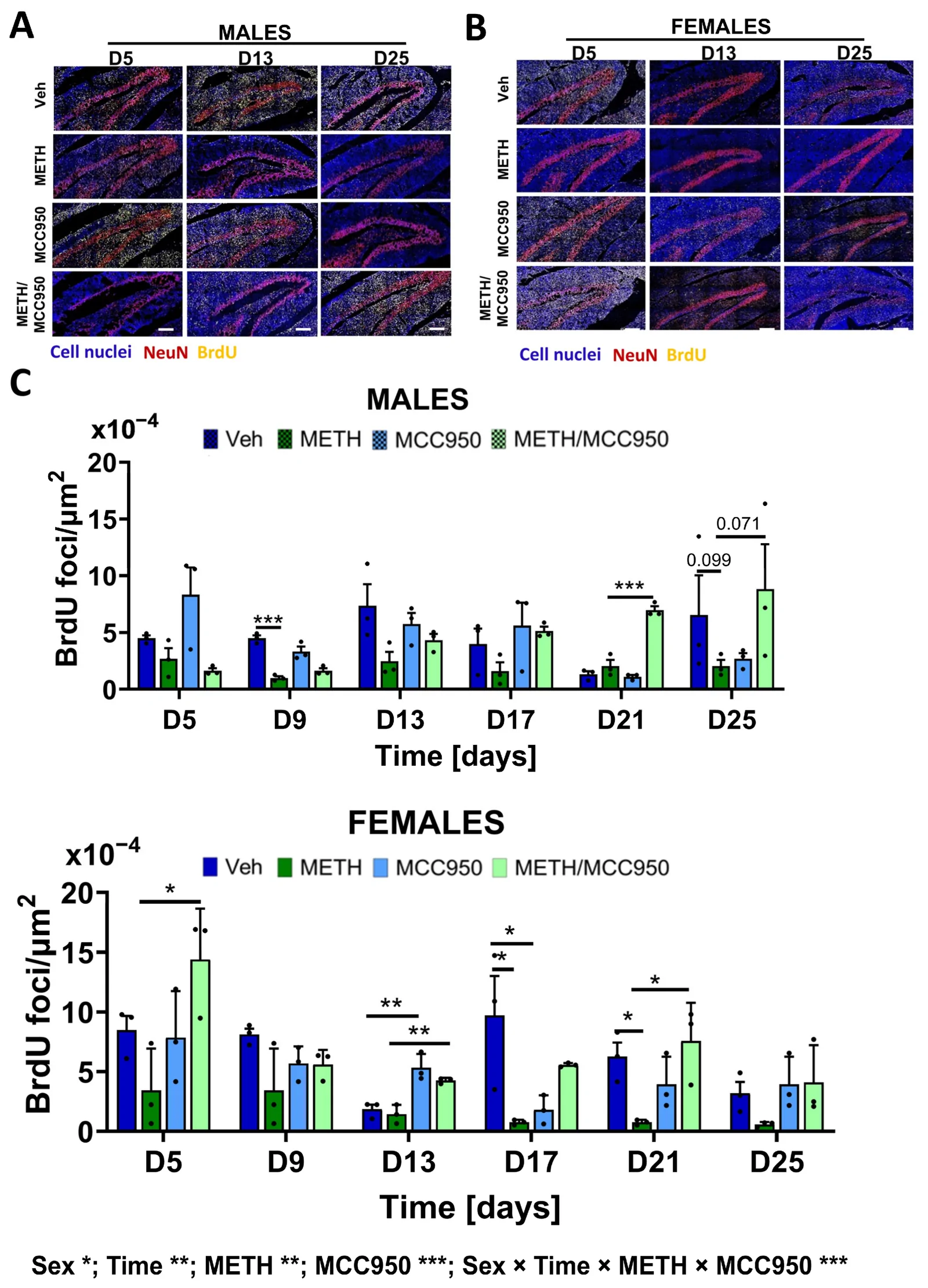
Impact of MCC950 administration on hippocampal cell proliferation in METH-exposed mice. Mice were treated with Veh, METH, MCC950, and/or METH/MCC950, as shown in Figure 1C. Representative immunostaining images from male (**A**) and female (**B**) mice at selected time points. Cell nuclei (DAPI) are visualized in blue, mature neurons (NeuN) in red, and proliferating cells (BrdU) in yellow; scale bar = 100 μm. (**C**) Quantitative analysis of BrdU-positive foci in the dentate gyrus across the experimental timeline in male and female mice. Data are presented as mean ± SEM. Overall effects of sex, time, METH, and MCC950, as well as their interactions, were assessed using four-way ANOVA. In addition, three-way ANOVA analyses were performed separately for each experimental day, followed by Tukey’s multiple-comparisons test; * *p* < 0.05, ** *p* < 0.01, and *** *p* < 0.001; *n* = 3 per each experimental group, three slices per mouse. Abbreviation: BrdU, 5-bromo-2′-deoxyuridine.

Elevated inflammatory responses can affect hippocampal neurogenesis [6]; therefore, we evaluated the impact of MCC950 on cell proliferation in the hippocampi of METH-exposed mice by using BrdU as a marker of replicating cells [45]. BrdU was administered to mice for five consecutive days during METH exposure, according to the diagram in Figure 1C. BrdU-positive foci were then quantified in the dentate gyrus of the hippocampi on D5 and then every fourth day until D25. Figure 5A,B show representative immunostaining images, while Figure 5C presents the quantitative analysis of BrdU-positive cells in male and female mice. Detailed information about all ANOVA tests performed is provided in Supplementary Table S3.

METH exposure was associated with alterations in hippocampal cell proliferation (*p* = 0.0038), with the magnitude of these changes differing over time (*p* = 0.0030) and between males and females (*p* = 0.0225). In male mice, METH treatment was accompanied by an overall tendency toward reduced numbers of BrdU-positive cells compared to vehicle-treated controls, with greater variability observed at individual time points. In female mice, METH administration similarly resulted in lower levels of cell proliferation. However, the pattern of these changes was more consistent over time. Importantly, hippocampal cell proliferation did not differ significantly across individual time points in vehicle-treated male or female mice (Supplementary Figure S3).

Treatment with MCC950 modified the effects of METH on hippocampal cell proliferation in both sexes, as indicated by a significant METH × MCC950 interaction (*p* < 0.0001). In male mice, the co-administration of MCC950 partially counteracted the METH-associated reduction in BrdU-positive cells, with the differences between the METH and METH/MCC950 groups becoming more pronounced at later stages of the experiment (D21 and D25). In female mice, the protective effect of MCC950 was evident across the experimental timeline, with MCC950-treated animals consistently exhibiting higher numbers of proliferating cells compared to the METH-only group.

Overall, these findings indicate that treatment with MCC950 mitigates METH-associated disturbances in hippocampal cell proliferation in a sex-dependent manner, as supported by the significant interactions involving the sex, time, and METH/MCC950 treatment factors.

**Figure 6:**
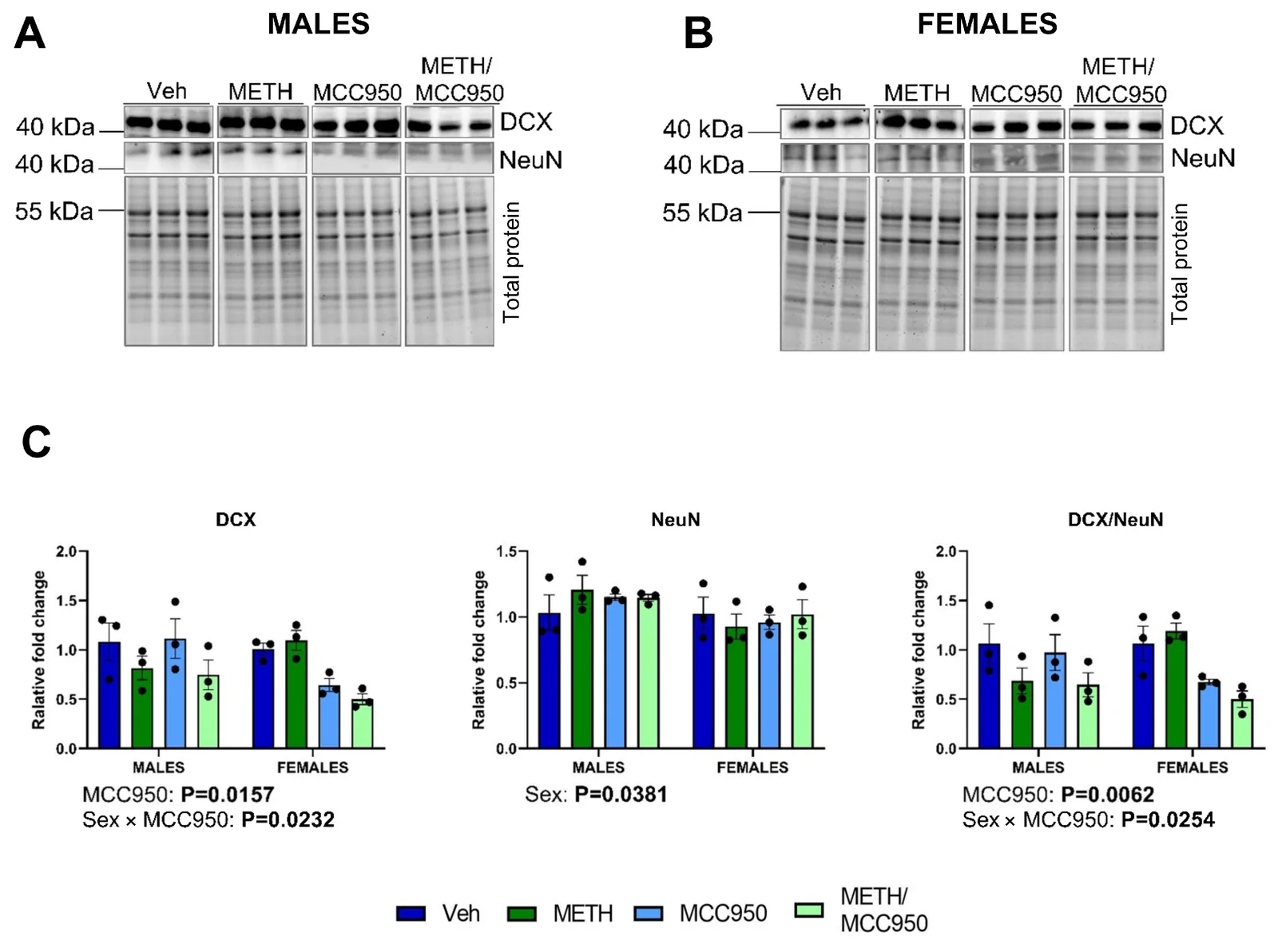
Impacts of METH and/or MCC950 administration on neuronal differentiation. Mice were exposed to METH and/or MCC950 for 5 days, and analyses were performed on the hippocampi collected on day 11, as visualized in Figure 1B. Homogenates from dissected hippocampi were analyzed via immunoblotting to assess neuronal differentiation by the evaluation of DCX and NeuN protein levels. Representative blots for male (**A**) and female (**B**) mice. (**C**) Quantitative results for males and females. Graphs represent the mean ± SEM. Statistical analysis was performed using a three-way ANOVA; *n* = 3 per experimental group. Abbreviations: DCX, doublecortin; NeuN, neuronal nuclear antigen.

Proliferating cells in the hippocampus that are critical for neurogenesis include neural progenitor cells (NPCs), which differentiate to mature neurons. Therefore, we evaluated neuronal differentiation by assessing DCX (doublecortin) and NeuN (neuronal-specific nuclear protein) levels via immunoblotting in male (Figure 6A) and female (Figure 6B) mice. DCX is a marker of immature neurons, whereas NeuN identifies mature neurons. We did not observe an interaction between METH treatment, MCC950, and sex in the three-way ANOVA (Figure 6C). However, the expression of DCX (*p* = 0.0157) and the ratio of DCX/NeuN (*p* = 0.0062) was affected by treatment with MCC950. Detailed statistical data are presented in Supplementary Table S3.

**Figure 7:**
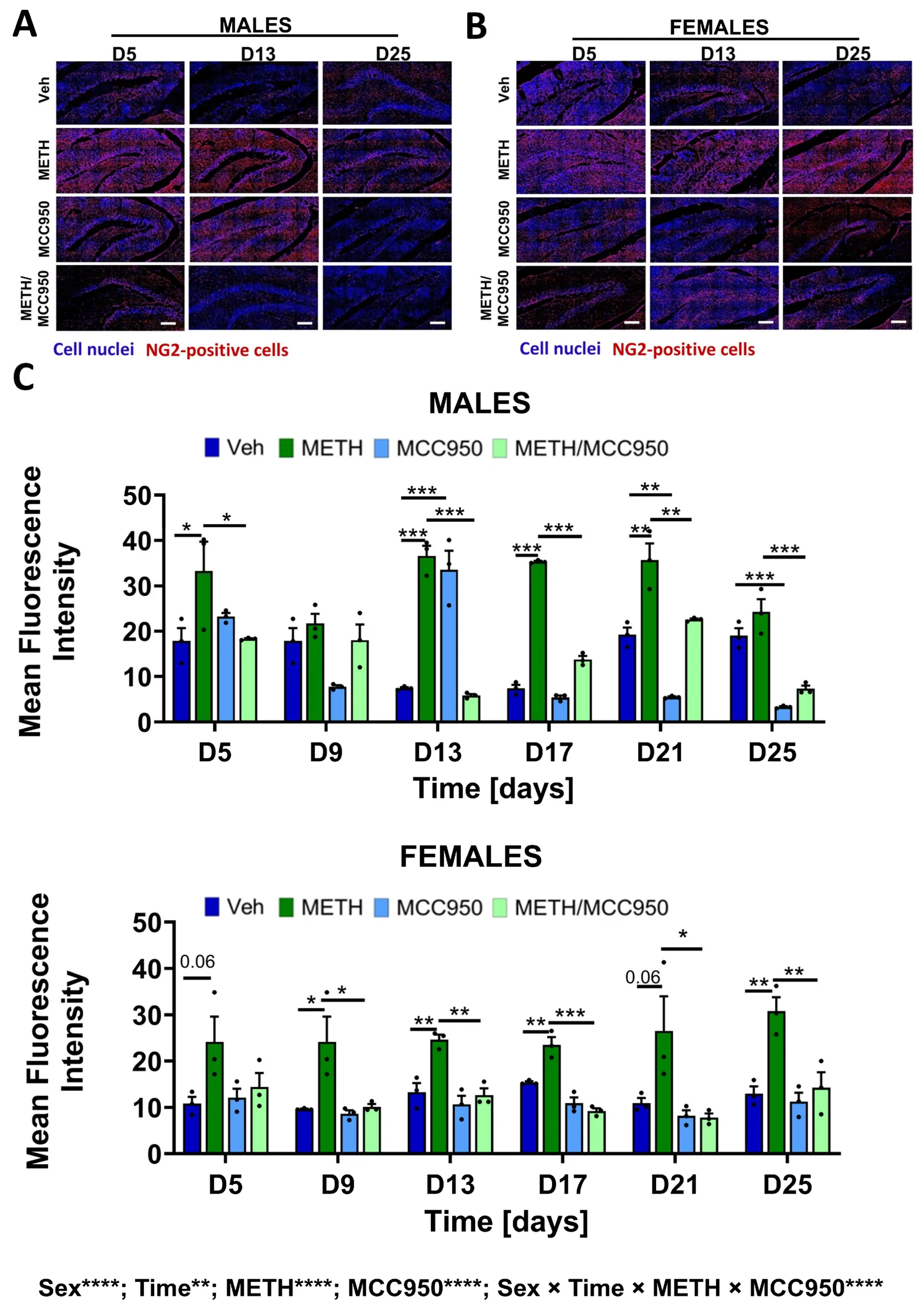
Impacts of METH and/or MCC950 administration on hippocampal NG2 immunoreactivity. Mice were treated with METH and/or MCC950, as shown in Figure 1C. Representative immunostaining images of the hippocampus from male (**A**) and female (**B**) mice at selected time points. Cell nuclei (DAPI) are labeled in blue, and NG2-positive cells are labeled in red; scale bars = 100 μm. (**C**) Quantitative analysis of NG2 immunoreactivity in male and female mice across the experimental timeline. Data are presented as mean ± SEM. Overall effects of sex, time, METH, and MCC950, as well as their interactions, were assessed using four-way ANOVA. In addition, three-way ANOVA analyses were performed separately for each experimental day, followed by Tukey’s multiple-comparisons test: * *p* < 0.05, ** *p* < 0.01, *** *p* < 0.001, and **** *p* < 0.0001, *n* = 3 per each experimental group, three slices per mouse. Abbreviation: NG2, neural/glial antigen 2.

We also evaluated the expression of NG2-positive cells, which are considered oligodendrocyte progenitor cells (OPCs) and ependymal cells and are known to become activated in the brain during pro-inflammatory processes [46]. METH exposure significantly increased NG2 immunoreactivity in the hippocampus (*p* = 0.000001). Statistically significant differences between the METH and Veh groups were observed in both male and female mice. Moreover, treatment with MCC950 protected against this effect (*p* = 0.000001). In both sexes, statistically significant differences between the METH/MCC950 and METH groups were detected at three out of the six analyzed time points (Figure 7A–C). Detailed data regarding the statistical tests performed, including exact *p*-values, are provided in Supplementary Table S3.

Additional one-way ANOVA analyses revealed time-dependent variability in NG2 immunoreactivity in vehicle-treated males (*p* < 0.001). In contrast, the NG2 levels in vehicle-treated females remained consistent over time (*p* = 0.08), supporting the robustness of the observed treatment effects in this sex (Supplementary Figure S4).

### 3.5. Impacts of METH and/or MCC950 on Hippocampal Proteome

**Figure 8:**
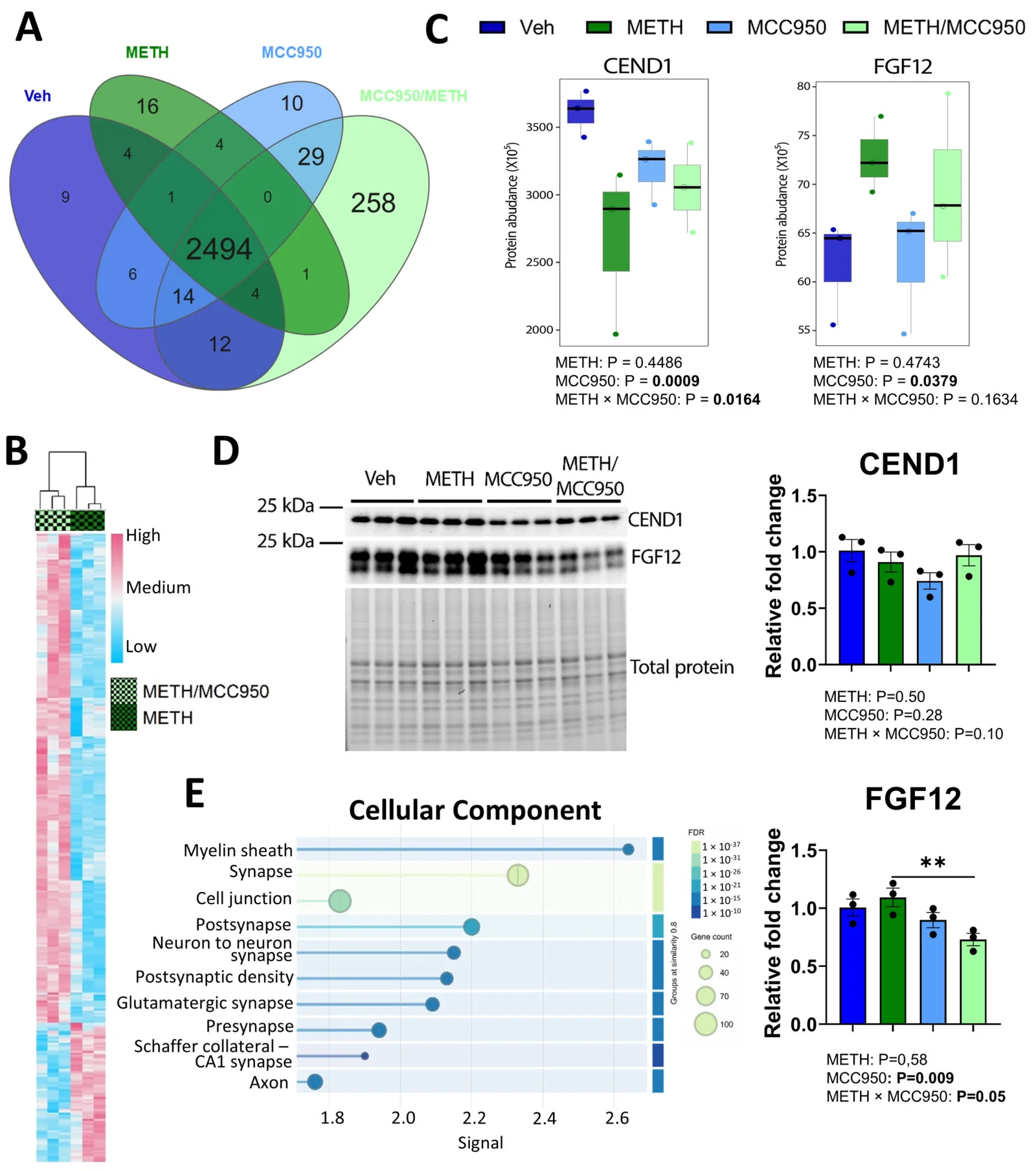

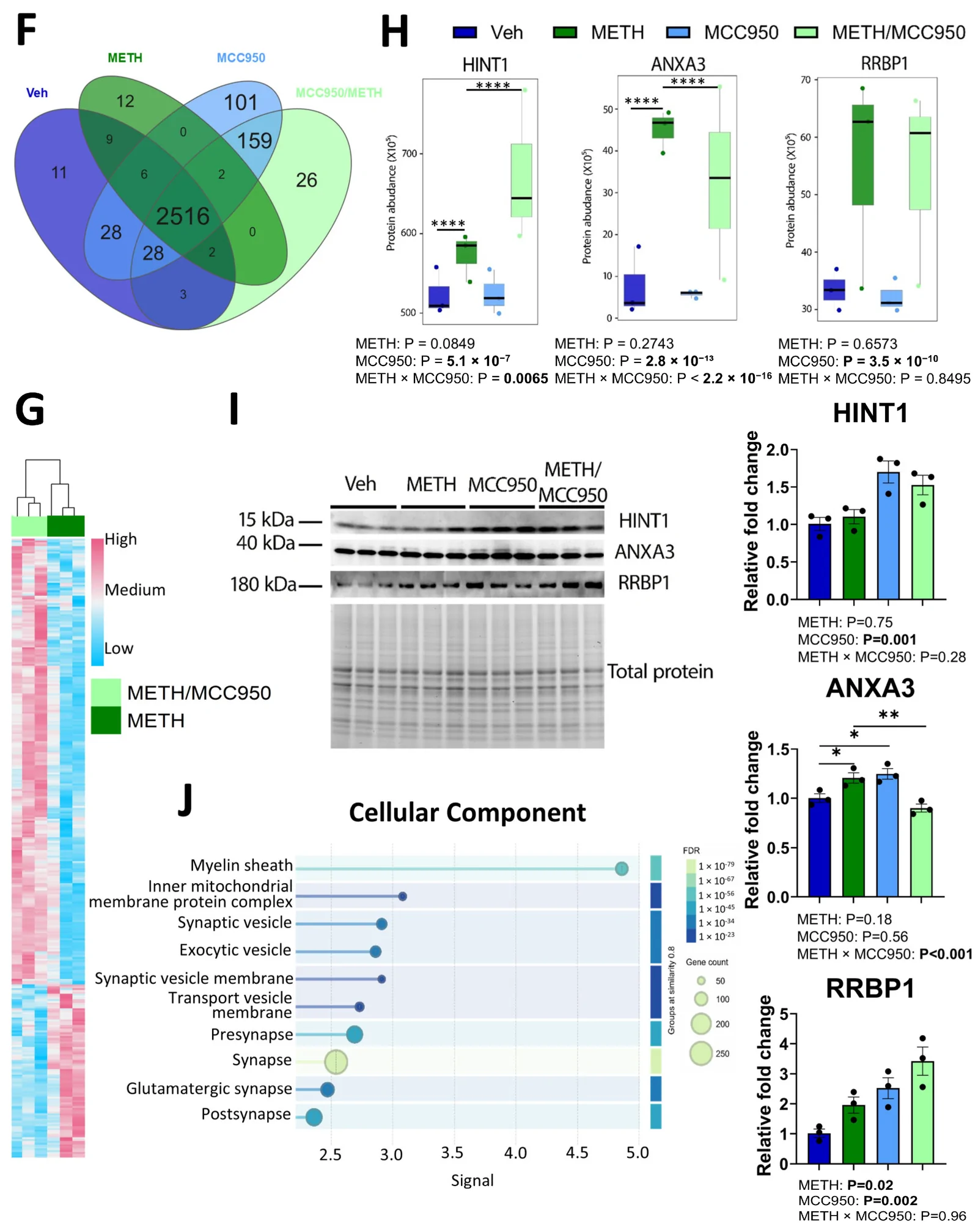
Impacts of METH and/or MCC950 administration on the hippocampal proteome. Homogenates from dissected hippocampi were analyzed by high-resolution mass spectrometry to assess the proteomic profiles of male (**A**–**E**) and female (**F**–**J**) mice. Proteins differentiating the METH and METH/MCC950 groups are represented as heatmaps (**B**,**G**). Based on the analyzed proteomics data, two proteins were selected in male mice (**C**) and three in female mice (**H**) for additional verification by immunoblotting (**D**,**I**). Proteins upregulated in the METH/MCC950 group relative to the METH group were subjected to GO term analysis in male (**E**) and female (**J**) mice. Data are presented as box plots showing median, minimum, and maximum values or as bar graphs representing the mean ± SEM. Data presented as box plots were analyzed using two-way ANOVA followed by Tukey’s multiple-comparisons test, whereas bar graph data were analyzed using a robust two-way ANOVA. Post hoc comparisons were conducted using robust, bootstrap-based tests on medians. * *p* < 0.05, ** *p* < 0.01, **** *p* < 0.0001; *n* = 3 per experimental group. Abbreviations: CEND1, cell cycle exit and neuronal differentiation protein 1; FGF12, fibroblast growth factor 12; HINT1, histidine triad nucleotide-binding protein 1; ANXA3, annexin A3; RRBP1, ribosome-binding protein 1.

A proteomic analysis using high-resolution mass spectrometry was performed to assess the effects of METH and MCC950 on the hippocampal protein profile (Figure 8). Overall, we detected 3061 hippocampal proteins in male mice and 3060 hippocampal proteins in female mice. Among them, 567 proteins in male (Supplementary Table S4) and 544 proteins in female mice (Supplementary Table S5) were detected only in a minority of samples (less than 50% in each group) and were excluded from quantitative analyses. The remaining 2494 hippocampal proteins in male mice (Supplementary Table S6) and 2516 hippocampal proteins in female mice (Supplementary Table S7) were identified in at least 50% of samples per group and were subjected to quantitative and binary analyses. The number of proteins unique to each experimental group and the number of proteins overlapping between experimental groups are provided in Figure 8A for male and in Figure 8F for female mice. The identities of these proteins are provided in Supplementary Tables S6 and S7. There were 314 proteins in male mice (Figure 8A,B) and 336 proteins in female mice (Figure 8F,G) that were differentially expressed when comparing the METH/MCC950 and METH groups.

The overall proteome profiles were distinctly different between the METH and METH/MCC950 groups in both male and female mice. Specifically, a higher number of proteins was upregulated in mice exposed to METH in combination with MCC950 as compared to METH alone. We then focused on two hippocampal proteins in male mice (CEND1 and FGF12; Figure 8C) and three hippocampal proteins in female mice (HINT1, ANXA3, and RRBP1; Figure 8H) whose levels were altered in the METH and METH/MCC950 groups (increase: FGF12, HINT1, ANXA3, and RRBP1; decrease: CEND1) compared to the Veh group. Moreover, our protein selection was based on the trend analysis using the Jonckheere–Terpstra test (FGF12, *p* = 0.0165; CEND1, *p* = 0.0590; HINT1, *p* = 0.0440; ANXA3, *p* = 0.0078; and RRBP1, *p* = 0.0115), robust two-way ANOVA, effect size analysis, and data from the literature indicating the associations of these proteins with neuroinflammation and the neuronal cell differentiation process. We then performed immunoblotting analyses (males, Figure 8D; females, Figure 8I) to confirm the proteomic results. In general, immunoblotting revealed similar trends of change as in the proteomic analyses, except for ANXA3 in female mice. The levels of this protein were decreased in immunoblotting (*p* = 0.003) but were elevated in proteomics in the METH/MCC950 group compared to the METH group.

We also examined the proteins of cellular components that were upregulated in the METH/MCC950 group relative to the METH group in male (Figure 8E) and female (Figure 8J) mice. The top upregulated proteins were associated with, among others, the myelin sheath, synapses, glutamatergic synapses, and pre-synapses, i.e., components that are involved in neuronal network formation.

### 3.6. Impact of MCC950 on Anxiety-like Behavior of METH-Administered Mice

**Figure 9:**
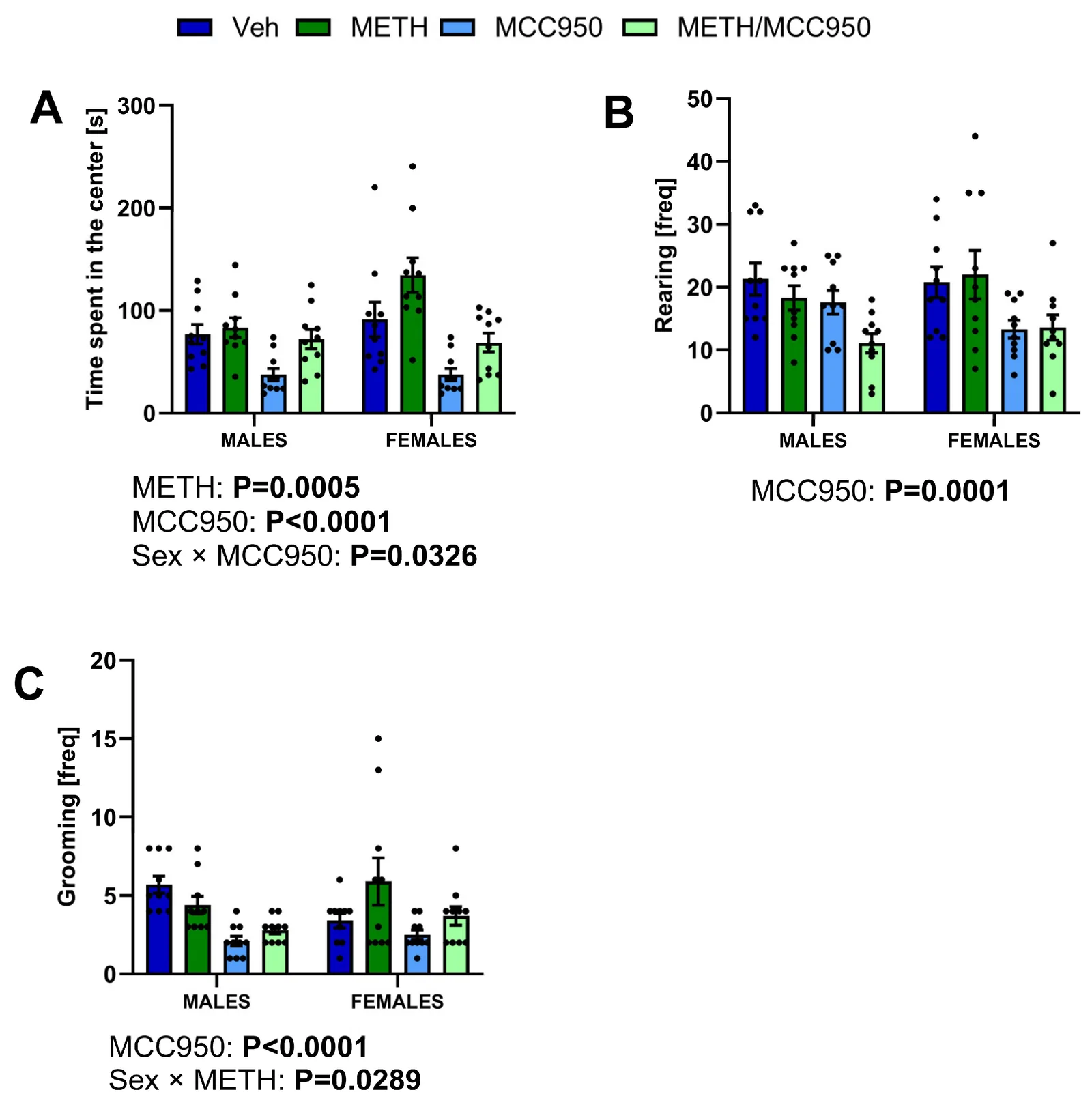
Impacts of METH and/or MCC950 administration on anxiety-like behavior. (**A**) Anxiety-like behavior as measured by the open field test in male and female mice. Alterations in exploratory (**B**) and repetitive behaviors (**C**). Graphs represent the mean ± SEM. Statistical analysis was performed using a three-way ANOVA; *n* = 10 per each experimental group.

In the final series of experiments, we evaluated whether the MCC950-mediated protective effect on METH-induced neurotoxicity and aberrant neurogenesis could also impact METH-mediated anxiety and cognitive decline. The open field test (OFT) was performed to evaluate anxiety-like behavior and locomotor activity. Reduced time spent in the center of the field during the OFT was considered an indicator of anxiety-like behavior. The administration of METH as well as MCC950 induced changes in anxiety-like behavior. A three-way ANOVA analysis revealed a significant effect of METH administration (*p* = 0.0005) on the time spent in the center of the open field. We also found significant effects of MCC950 administration in both male and female mice (*p* < 0.0001), as well as an interaction between MCC950 and sex (*p* = 0.0326) (Figure 9A). However, we did not observe any significant interaction between METH, MCC950 administration, and sex in the three-way ANOVA.

Two additional indicators of anxiety-like symptoms, namely vertical activity and reduced grooming frequency, also suggested an influence on anxiety following MCC950 treatment. Indeed, mice exposed to MCC950 showed a reduction in exploratory behavior. A three-way ANOVA revealed a significant effect of MCC950 treatment on the vertical activity of mice as assessed by the rearing frequency (*p* = 0.0001) (Figure 9B). MCC950 administration also resulted in changes in repetitive behavior as expressed by a reduced grooming frequency (*p* < 0.0001) (Figure 9C). A significant interaction of METH x sex was revealed in the three-way ANOVA on the grooming frequency (*p* = 0.0289). However, we did not find significant effects of sex, METH, or the interaction of these factors.

The administration of METH and/or MCC950 did not alter spontaneous locomotor activity as assessed by the total distance moved and velocity in both sexes (for details, please see Supplementary Figure S5 and Table S4).

### 3.7. Impacts of MCC950 on Recognition Memory and Spatial Learning in METH-Administered Mice

To evaluate the impacts of MCC950 treatment on hippocampal-dependent cognitive functions, such as learning and memory, we performed the NOR and MWM tests. In the NOR test, the % time spent with a novel object was analyzed, which represents recognition memory sensitivity. A three-way ANOVA revealed no significant effects of METH or MCC950 administration. However, we observed a significant effect of sex on the % time spent with the novel object (*p* = 0.0025), suggesting sex differences in response to the used treatments on recognition memory. Mice in the METH- and MCC950-treated groups did not spend significantly more time exploring the novel object than the familiar one, suggesting that non-spatial memory was not affected by any of the employed treatments (Supplementary Figure S6).

**Figure 10:**
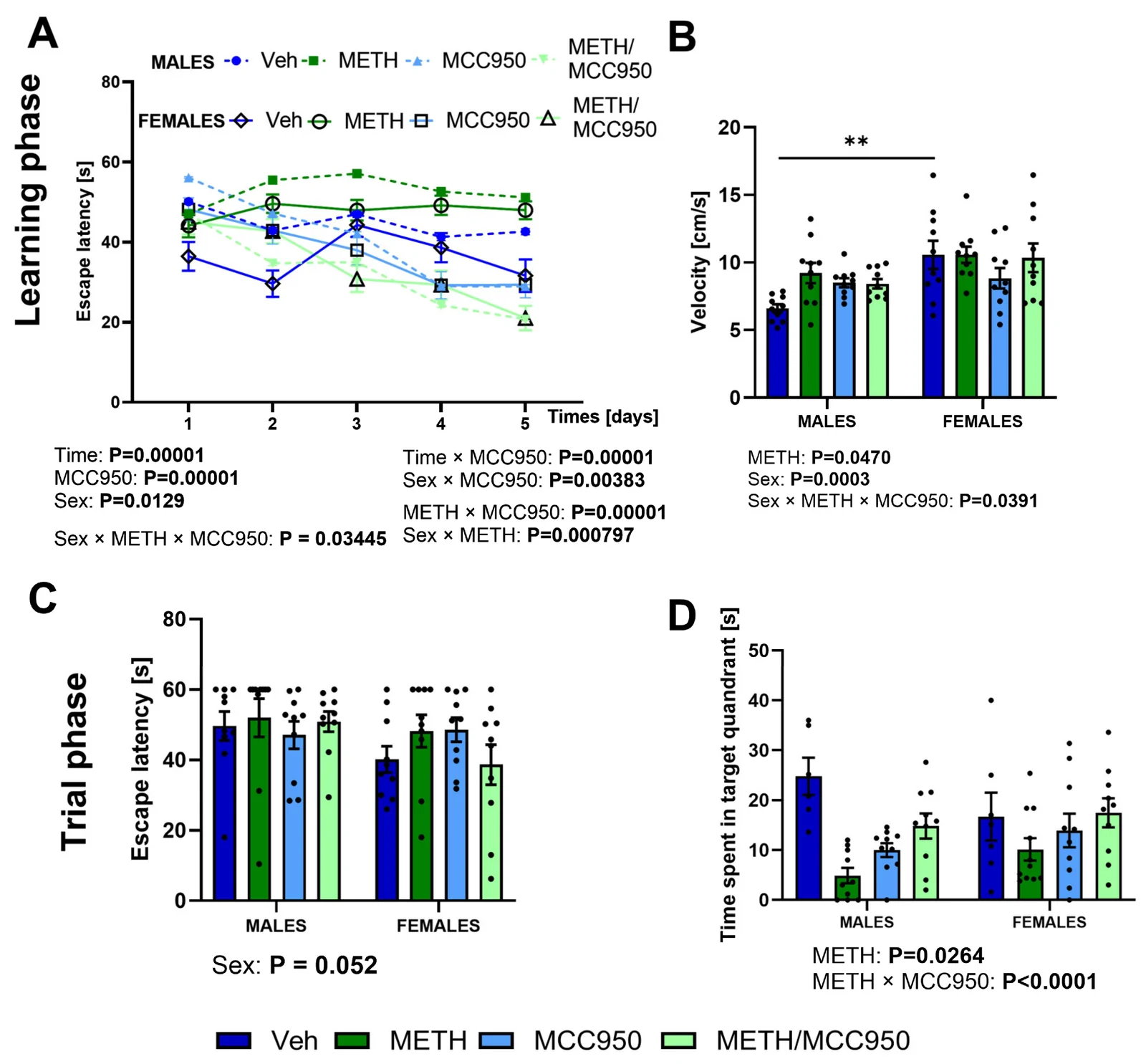
Assessment of METH and/or MCC950 administration effects on learning and spatial memory. Mice were subjected to METH and MCC950 administration as shown in Figure 1C, followed by measurements of escape latency (**A**,**C**), velocity (**B**), and time spent in the target quadrant (**D**). Measurements were performed during the learning phase (**A**,**B**) and the test trial phase (**C**,**D**). Graphs represent the mean ± SEM. Statistical analysis was performed using four- (**A**) and three-way ANOVA (**B**–**D**), followed by Tukey’s multiple-comparisons test. Statistically significant differences are marked as follows: ** *p* < 0.01; *n* = 10 per each experimental group.

Next, we employed the MWM test to assess cognitive function (Figure 10). We observed a significant effect of time (*p* = 0.00001), MCC950 administration (*p* = 0.00001), sex (*p* = 0.0129), and their interactions (time × MCC950: *p* = 0.00001, sex × MCC950: *p* = 0.00383) on the escape latency parameter. During the first five days of the MWM assessment (learning phase, D1–D5), mice from the MCC950 and METH/MCC950 groups exhibited an improvement in learning the location of the platform (METH × MCC950: *p* = 0.00001). The administration of MCC950 also improved the learning of the platform position in METH-exposed female mice (sex × METH × MCC950: *p* = 0.03445, sex × MCC950: *p* = 0.00383, sex × METH: *p* = 0.000797) (Figure 10A).

There was a significant effect of METH administration (*p* = 0.0470), sex (*p* = 0.0003), and the interaction of METH, MCC950, and sex (*p* = 0.0391). When analyzing the velocity during the learning phase of the MWM test, female mice from the vehicle group exhibited a greater velocity as compared to the vehicle group of males, following a post hoc test (*p* = 0.0043) (Figure 10B).

During the probe trial of the MWM test, we found a significant effect of sex on the escape latency parameter (*p* = 0.052) (Figure 10C). A three-way ANOVA revealed that METH administration disrupted long-term spatial memory, which was reflected by a shorter period of time spent in the targeted quadrant (*p* = 0.0264). The administration of MCC950 modified these effects, suggesting a beneficial impact of NLRP3 inhibition on spatial learning. Indeed, METH/MCC950-treated male and female mice spent more time in the targeted quadrant of the water maze (METH × MCC950 interaction: *p* < 0.0001) (Figure 10D).

## 4. Discussion

The neuropharmacological impact of METH results in excessive dopamine release, leading to its oxidation and the formation of reactive oxygen species (ROS) [47], with secondary damage to proteins, lipids, and DNA in neurons and triggering an inflammatory cascade [48,49]. Ultimately, METH use reduces the number of dopaminergic terminals and disrupts cognitive, emotional, and motor functions, mirroring the changes observed in the early stages of neurodegenerative diseases [50,51]. While METH is recognized as a pro-inflammatory agent, the role of inflammasomes in METH-induced neurotoxicity is not fully understood. Therefore, we evaluated the impacts of METH exposure on the inflammasome profile in mouse hippocampi via the assessment of pattern recognition receptor (PRR) proteins, which assemble inflammasomes and respond to cellular distress. We identified NLRP3 and NLRP1 as inflammasomes with strong tendencies to be upregulated in response to METH treatment, supporting some earlier observations [49]. There was also interesting sexual dimorphism, as METH’s tendency to upregulate proteins associated with inflammasome formation was more pronounced in female mice. This observation corresponds with a previous report indicating that NLRP1 levels in neurodegenerative conditions were higher in female than male brains [52].

Due to the role of the NLRP3 inflammasome in METH-induced neurotoxicity, we next employed MCC950, an inhibitor of the canonical and non-canonical activation of this inflammasome. Consistent with the upregulation of the inflammasomes, we observed an increase in inflammatory cytokines in the serum of both male and female mice exposed to METH, followed by efficient attenuation after the administration of MCC950. These systemic pro-inflammatory effects of METH may be caused by its direct toxic impact, as well as by disturbances of the intestinal barrier and liver [2,53]. In contrast to serum, a different pattern of METH-induced inflammatory responses was observed in the hippocampi of male and female mice. Specifically, METH administration upregulated the expression of twelve pro-inflammatory cytokines in the hippocampi of male mice, and none of these cytokines responded to MCC950 treatment. In female mice, METH-induced cytokine level changes were limited to the upregulation of IL-12(p70) and TNF-α. Moreover, their levels normalized following MCC950 administration. The observed sexual dimorphism may be related to higher estrogen levels in female mice, which may increase their neuroprotective and anti-inflammatory abilities by inhibiting microglial activity or suppressing the production of pro-inflammatory cytokines [54]. In contrast, elevated testosterone levels in male mice have been linked to higher TLR4 expression and increased stimulation of the NF-κB pathway, contributing to the so-called pro-inflammatory brain phenotype [55].

METH induces the redistribution of occludin in brain endothelial cells from plasma membranes to endosomes via the activation of the actin-related protein 2/3 (Arp2/3) complex [56]. Previous experimental studies showed that MCC950 administration preserved the integrity of the BBB by reducing hippocampal hyperpermeability and restoring the expression of TJ proteins like claudin-5 and ZO-1 in neuroinflammatory and ischemic conditions [57,58,59]. Moreover, MCC950 blocked caspase-1 and MMP-9 activity, preventing the downregulation of TJs in brain microvascular endothelial cells [60]. In the present study, the increased TJ expression following treatment with MCC950 was consistent with these results and may reflect the restoration of cellular barrier integrity. Moreover, MCC950 affected ZO-1, claudin-5, and occludin in a sex-dependent manner. These results are consistent with sex differences in the expression of BBB-related genes, including occludin and claudin-5 [61,62,63]. Experimental evidence of sex differences in NLRP3 inflammasome activation and inhibition have been reported across diverse systemic conditions with a neurological impact (as reviewed in [64]). The main producers of inflammatory mediators in the CNS are activated microglial cells, which are known to exhibit significant sexual dimorphism. Sex differences in immune reactivity and the metabolism of microglial cells across the lifespan [65] have been linked to sexual dimorphism in Alzheimer’s disease [66], response to CNS injury and stroke [67], and even aging [68]. Thus, sex-dimorphic microglia rewiring in inflammatory conditions could also have contributed to the differences in METH-induced inflammatory responses in male and female mice that were observed in the present study.

Inflammatory processes are known to affect the proliferation and differentiation of progenitor cells; therefore, these events were also evaluated. We observed a protective effect of MCC950 administration on METH-induced alterations of hippocampal cell proliferation. Specifically, METH exposure decreased cell proliferation, which was attenuated by treatment with MCC950. These effects were observed in both male and female mice, even though their kinetics were different. The impact of METH administration on cell proliferation was reported previously [7,21]. While the current report links these effects, at least in part, to inflammasome activation, we cannot exclude the possibility that the reduction in hippocampal cell proliferation may be the effect of general oxidative stress induced by METH.

METH administration diminished the ratio of immature to mature neurons and showed a strong tendency to decrease the number of DCX-positive immature neurons, but only in male mice. Moreover, MCC950 treatment attenuated these effects. Similar observations have been made in studies investigating the effects of inflammation following focal cerebral ischemia. The use of MCC950 after ischemic injury yielded beneficial effects, including an increased number of NeuN-positive cells [69]. In female mice, METH administration did not affect the numbers of immature and mature neurons, illustrating sexual dimorphism and protection against METH-induced neurotoxicity. Surprisingly, treatment with MCC950 alone and in combination with METH significantly decreased the number of DCX-positive immature neurons and the ratio of immature to mature neurons, suggesting off-target toxic effects. In contrast to the diminished cell proliferation and neuronal differentiation, METH exposure increased the number of NG2-positive cells, which are identified as progenitor cells, oligodendrocytes, pericytes, and Schwann cells. These results are consistent with previous reports of enhanced NG2 immunoreactivity following METH exposure [46,70].

Due to the significant impact of METH on the hippocampus, we assessed the hippocampal proteomic profile. Interestingly, markedly more upregulated proteins were determined in mice exposed to METH plus MCC950 when compared to METH alone. Proteins of interest in male mice were CEND1 and FGF12, which were downregulated and upregulated by METH exposure, respectively. However, treatment with MCC950 attenuated these effects. CEND1 is directly involved in neuronal differentiation, and its reduced level causes a delay in neuronal maturation [71]. On the other hand, FGF12 acts intracellularly, regulating sodium channels and neuronal excitability, as observed in the early phase of METH treatment [72,73]. In female mice, identified proteins of interest were HINT1, ANXA3, and RRBP1, which were upregulated by METH exposure. An elevated level of HINT1 was noted during the acquisition phase of METH addiction [74]. An increase in ANXA3 levels was reported during microglial activation [75]. RRBP1 was observed to be induced by endoplasmic reticulum (ER) stress and functions as an adaptive factor, supporting cell survival during the stress response [76,77]. In our study, treatment with MCC950 did not affect the METH-induced upregulation of HINT1, ANXA3, and RRBP1, suggesting an NLRP3-independent mechanism.

The upregulation of inflammatory responses and aberrant cell proliferation and neurogenesis may have negative effects on anxiety and cognitive function. It is worth noting that anxiety- and depressive-like symptoms are common symptoms in METH use disorder, especially in the acute withdrawal period [78,79,80]. Therefore, we evaluated the impacts of METH and/or MCC950 on mouse anxiety and cognition. Surprisingly, MCC950-treated female mice exhibited thigmotaxis behavior by avoiding the central part of the field in the OFT. In contrast, MCC950-treated male mice diminished their grooming and rearing, which might reflect displacement behavior or a general reduction in self-soothing behaviors due to elevated stress [81]. These different displays of anxiety-like symptoms in male and female mice illustrate sex-dependent responses to MCC950-mediated effects. Moreover, they confirm the potential off-target behavioral effects of this small molecule. Our observations are in contrast to previous reports [82,83,84] describing the alleviation of depression-like behavior in male mice following treatment with MCC950. In our study, we did not observe locomotor activity changes following METH administration, which has also been reported in the literature [7,85,86]. While the acute administration of METH causes locomotor hyperactivity [87], our open field test was performed three days after completing METH administration, i.e., during the withdrawal phase.

We also observed that METH exposure degraded spatial memory and learning abilities. These results are consistent with studies demonstrating profoundly impaired spatial memory in male rats [88] and mice [89] following METH administration. Importantly, treatment with MCC950 resulted in protection against METH-induced spatial memory deficits in both sexes. Mechanistically, these improved learning abilities are likely to be caused by the attenuation of inflammasome-mediated neuroinflammation and neurodegeneration. MCC950 also decreased mature IL-1β expression mediated by caspase 1 in the cerebella of METH-exposed mice, which was associated with neuroprotective effects [34]. Overall, these results suggest that MCC950 can attenuate METH-induced cognitive impairment by mitigating aberrant neurogenesis and suppressing the neuroinflammation process.

Our important findings reveal a mechanistic dissociation between cytokine levels and behavioral outcomes in METH-exposed mice. For example, the administration of MCC950 resulted in improved spatial learning in male mice despite the persistent elevation of most METH-induced hippocampal cytokines. This indicates that cytokine levels may not be a reliable determinant of behavioral outcomes. It has been reported that cytokines exert context-dependent effects on neuronal and microglial function, and their elevation does not directly transfer to functional outcomes [90,91,92]. Our proteomic data, showing the broad remodeling of synaptic and structural proteins, also support a circuit-level mechanism rather than purely cytokine-driven cognitive alterations. MCC950 can engage additional molecular targets, including carbonic anhydrase 2 [93], which may lead to behavioral improvements in the presence of persistent cytokine abnormalities. Overall, our results demonstrate broader neuroimmune–metabolic modulation by MCC950 and suggest that the reversal of METH-induced cognitive impairment may not require the normalization of hippocampal cytokine levels.

Study limitations. The analyses in this study were conducted on hippocampal homogenates, which did not allow us to assess the contributions of specific cell types to METH toxicity. In fact, several METH-induced effects could result from the direct activation of astrocytes and/or microglial cells. Although MCC950 demonstrated neuroprotective effects in our study, its potential risks and limitations should be acknowledged. MCC950 induced anxiety-like behavior in female mice and reduced grooming in males, indicating that this small molecule can negatively modulate affective and stress-related responses in a sex-dependent manner. Moreover, MCC950 appeared to reduce the number of DCX-positive immature neurons in female mice, suggesting potential interference with the early stages of hippocampal neurogenesis even in the absence of METH exposure. Although these findings are based on a relatively small sample size and should be interpreted cautiously, they indicate that NLRP3 inhibition may exert sex-dependent impacts on neurogenic processes. Finally, MCC950 did not improve recognition memory in the NOR test, underscoring that its beneficial effects on cognition may be domain-specific rather than universal. Treatment with both METH and MCC950 can affect the acid–base balance in the brain, which could contribute to the observed toxicity and off-target effects of MCC950.

An additional limitation concerns the estrous cycle in female mice, which represents a well-recognized potential confounder in behavioral research. Fluctuating ovarian hormone levels across the cycle can modulate a wide range of non-sexual behaviors, including emotionality, cognition, and drug-related responses [94,95,96]. However, growing evidence suggests that, although the estrous cycle influences specific neural circuits, its contribution to overall behavioral variability is often comparable to that observed in males [97]. Nonetheless, the impact of ovarian hormones should be taken into account when interpreting the sex-dependent behavioral outcomes of the present study.

These limitations align with concerns that are increasingly reported in the current literature. In an interventional model of diabetic kidney disease, chronic MCC950 treatment unexpectedly aggravated renal inflammation and fibrosis, leading to mesangial expansion and glomerulosclerosis [98]. Moreover, the clinical development of MCC950 and related diarylsulfonylurea NLRP3 inhibitors has been discontinued due to hepatotoxicity, and recent reviews emphasize drug-induced liver injury as a major obstacle for this compound class [99]. Proteome-wide profiling further indicates that MCC950 interacts with additional cellular targets, which may contribute to both efficacy and toxicity beyond canonical NLRP3 blockade [93].

Several methodological considerations should also be noted when interpreting the results of the present study. We applied an experimental model reflecting the pattern of human METH intake, which was consistent with the translational aim of our research. This model is based on the tendency of METH-misusing individuals to continuously increase their doses of psychoactive substances to maintain comparable psychological and physical sensations [100]. However, the analyses reported in the present study were performed during METH withdrawal.

## 5. Conclusions

MCC950 holds preventive potential for mitigating the detrimental effects associated with exposure to METH. The administration of this small molecule produced beneficial outcomes at both the behavioral level, such as spatial learning, and the neurochemical level, namely suppressed activation of inflammatory processes and enhanced cell proliferation in the hippocampus. Notably, these effects were more pronounced in male mice, displaying sexual dimorphism. However, we also observed several off-target effects of this inhibitor. Overall, this study underscores the potential of targeting inflammasome activation as a preventive strategy against METH-induced neurotoxicity, while also highlighting the need for further in-depth investigations, including sex-dependent effects.

## Data Availability

The data presented in this study are openly available in the Proteo-meXchange Consortium repository at https://www.ebi.ac.uk/pride (accessed on 25 July 2025), reference number PXD066569.

## Supplementary Materials

The following supporting information can be downloaded at https://www.mdpi.com/article/10.3390/cells15161443/s1. Figure S1: Body weight and food intake in male and female mice; Figure S2: Serum AST and ALT levels following 5-day exposure to METH and MCC950; Figure S3: Hippocampal cell proliferation levels in vehicle-treated mice over the course of the experiment; Figure S4: Hippocampal NG2 immunoreactivity levels in vehicle-treated mice over the course of the experiment; Figure S5: METH and/or MCC950 administration did not change spontaneous locomotor activity assessed by total distance moved (A) or velocity (B) in both sexes in the Open Field test; Figure S6: Assessment of METH and/or MCC950 administration on recognition memory measured by the Novel Object test; Table S1: Evaluation of the estrous cycle in female mice; Table S2: Producers, catalog numbers of primary antibodies, blocking buffers, antibodies diluents, and dilutions of primary and secondary antibodies; Table S3: Statistical source data for the extended data from the ANOVA test; Table S4: Binary proteomic analysis of male samples; Table S5: Binary proteomic analysis of female samples; Table S6: Quantitative proteomic analysis of male samples; Table S7: Quantitative proteomic analysis of female samples.

## Abbreviations

The following abbreviations are used in this manuscript:

BBB	Blood–Brain Barrier
MWM	Morris Water Maze
NOR	Novel Object Recognition Test
NPC	Neural Progenitor Cell
OFT	Open Field Test
OPC	Oligodendrocyte Progenitor Cell
PFA	Paraformaldehyde
ROS	Reactive Oxygen Species
