# Supplementary material for: Inflammasome Inhibitor MCC950 Attenuates Methamphetamine-Induced Hippocampal Neurotoxicity and Aberrant Neurogenesis in a Sex-Dependent Manner
Source: Cells. 2026 Aug 11;15(16):1443. doi: 10.3390/cells15161443 (PMC13511100; doi:10.3390/cells15161443)
Supplement: Supplementary file 1 [file cells-15-01443-s001.zip › cells-4443480-supplementary.pdf]

Supplementary Materials

Inflammasome Inhibitor MCC950 Attenuates Methamphetamine-Induced Hippocampal Neurotoxicity and Aberrant Neurogenesis in a Sex-Dependent Manner

Mateusz Smolarz [1,2], Natalia Pondel [1], Gracjana Zając [1], Agata Kurczyk [3], Monika Pietrowska [2,4], Marta Gawin [2,4], Magdalena Dębiec [1], Andrzej Małecki [1], Marta Nowacka-Chmielewska [1,*,†] and Michal Toborek [1,5,*,†]

[1] Institute of Physiotherapy and Health Sciences, Academy of Physical Education, 40-065 Katowice, Poland; m.smolarz@awf.katowice.pl (M.S.); n.pondel@awf.katowice.pl (N.P.); gracjana.zajac@gmail.com (G.Z.); d201110@365.sum.edu.pl (M.D.); a.malecki@awf.katowice.pl (A.M.)

[2] Maria Sklodowska-Curie National Research Institute of Oncology, Gliwice Branch, 44-102 Gliwice, Poland; m.pietrowska@igbzpan.pl (M.P.); m.gawin@igbzpan.pl (M.G.)

[3] Department of Biostatistics and Bioinformatics, Maria Skłodowska-Curie National Research Institute of Oncology, Gliwice Branch, 44-102 Gliwice, Poland; agata.kurczyk@gliwice.nio.gov.pl

[4] Institute of Genetics and Animal Biotechnology of the Polish Academy of Sciences, 05-552 Jastrzebiec, Poland

[5] Department of Biochemistry and Molecular Biology, University of Miami Miller School of Medicine, Miami, FL 33136, USA

* Correspondence: m.nowacka@awf.katowice.pl (M.N.-C.); mtoborek@med.miami.edu (M.T.); Tel.: +48-32-207-52-48 (M.N.-C.); +1-305-243-0230 (M.T.)

† These authors contributed equally to this work.

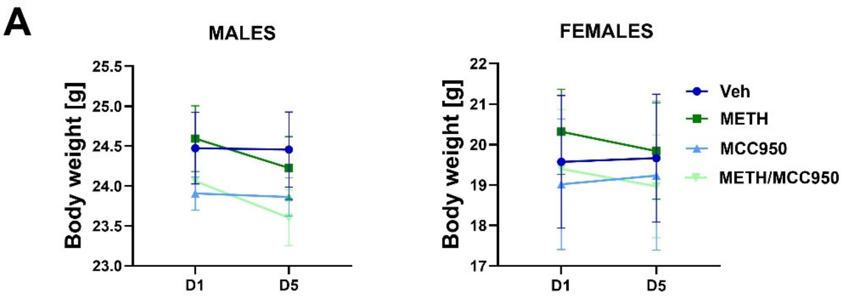

p-Value (three-way ANOVA)

	F (DFn, DFd), P value for Time factor	F (DFn, DFd), P value for MCC950 factor	F (DFn, DFd), P value for METH factor	F (DFn, DFd), P value for Time x MCC950 factor	F (DFn, DFd), P value for Time x METH factor	F (DFn, DFd), P value for MCC950 x METH factor	F (DFn, DFd), P value for Time x MCC950 x METH factor
**Males**	F (1, 21) = 30.80 **P<0.0001**	F (1, 21) = 3.402 P=0.0793	F (1, 21) = 0.0288 P=0.8667	F (1, 21) = 0.8807 P=0.3587	F (1, 21) = 20.76 **P=0.0002**	F (1, 21) = 1.810e -005 P=0.9966	F (1, 21) = 0.1513 P=0.7012
**Females**	F (1, 21) = 5.472 **P=0.0293**	F (1, 21) = 2.959 P=0.1001	F (1, 21) = 1.126 P=0.3006	F (1, 21) = 0.4941 P=0.4898	F (1, 21) = 43.69 **P<0.0001**	F (1, 21) = 0.9470 P=0.3416	F (1, 21) = 0.0927 4 P=0.7637

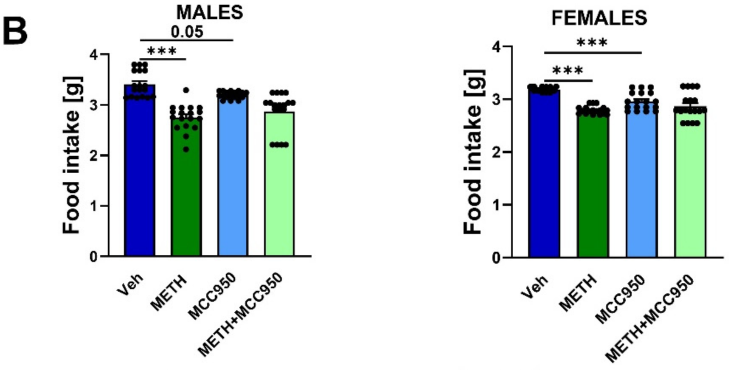

<u>two-way Anova</u>
METH: F (1, 68) = 61.10; **P<0.001**
MCC950: F (1, 68) = 0.6142; P=0.44
METH x MCC950: F (1, 68) = 7.037; **P=0.010**

<u>two-way Anova</u>
METH: F (1, 68) = 43.36; **P<0.001**
MCC950: F (1, 68) = 4.214; **P=0.04**
METH x MCC950: F (1, 68) = 15.86; **P<0.001**

Supplementary Figure S1. Body weight and food intake in male and female mice. Measurements were conducted on days D1 and D5 of the experiment in accordance with the experimental design shown in Figure 1. (A) Assessment of body weight in male and female mice. Data are presented separately for males and females. Statistical analysis was performed using three-way ANOVA; F and p values for main effects and interactions are shown below the graphs. (B) Food intake was quantified in male and female mice across treatment groups. Statistical analysis was performed using a two-way ANOVA followed by Šidák's multiple comparisons test. ***p < 0.001. Data are presented as mean ± SEM. In panel B, only between-group differences are shown (Veh vs. METH; Veh vs. MCC950; METH vs. METH/MCC950).

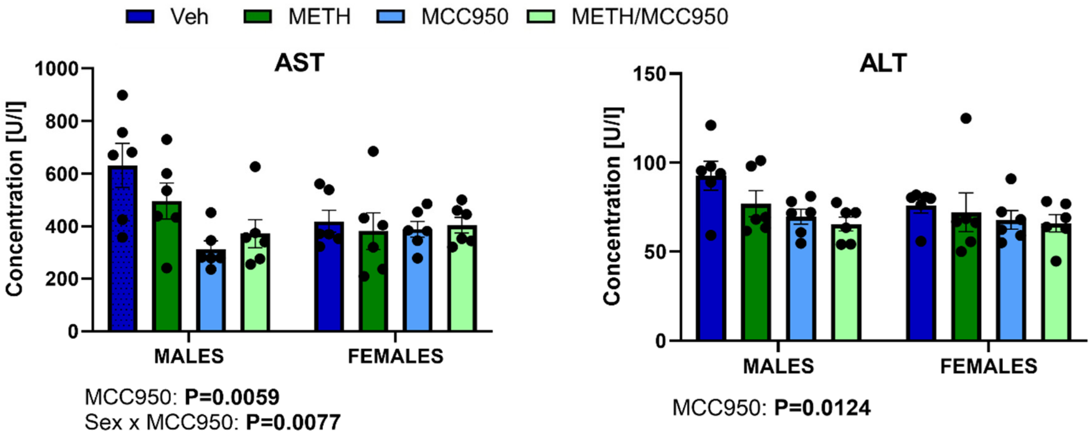

Supplementary Figure S2. Serum AST and ALT levels following 5-day exposure to METH and MCC950. Quantitative analysis of serum aspartate aminotransferase (AST) and alanine aminotransferase (ALT) levels in male and female mice. Data are presented as mean ± SEM. Statistical analysis was performed using three-way ANOVA. n = 6 per each experimental group.

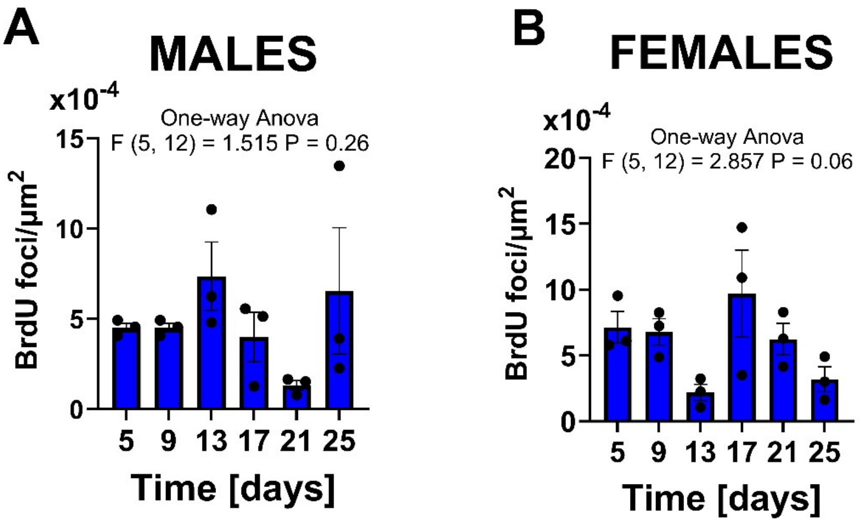

Supplementary Figure S3. Hippocampal cell proliferation levels in vehicle-treated mice over the course of the experiment. Quantitative analysis of BrdU-positive foci in the dentate gyrus of vehicle-treated male (A) and female (B) mice across the experimental timeline. Data are presented as mean ± SEM. Statistical analysis was performed using one-way ANOVA. n = 3 per each experimental group, three slices per mouse.

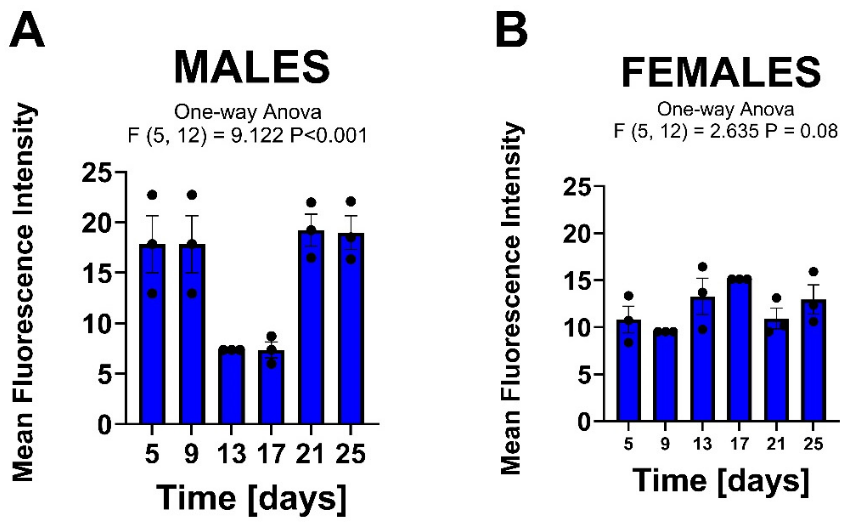

Supplementary Figure S4. Hippocampal NG2 immunoreactivity levels in vehicle-treated mice over the course of the experiment. Quantitative analysis of NG2 immunoreactivity in the hippocampus of vehicle-treated male (A) and female (B) mice across the experimental timeline. Data are presented as mean ± SEM. Statistical analysis was performed using one-way ANOVA. n = 3 per experimental group, three slices per mouse.

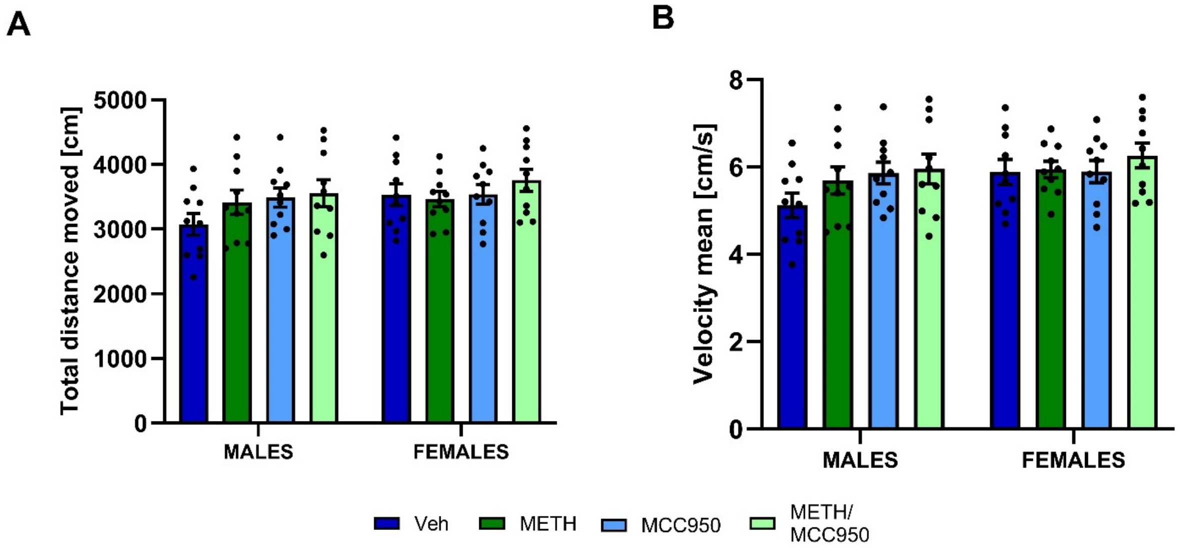

Supplementary Figure S5. METH and/or MCC950 administration did not change spontaneous locomotor activity assessed by total distance moved (A) or velocity (B) in both sexes in the Open Field test. Graphs represent the mean ± SEM (n = 10 per group). Statistical analysis was performed using a three-way ANOVA.

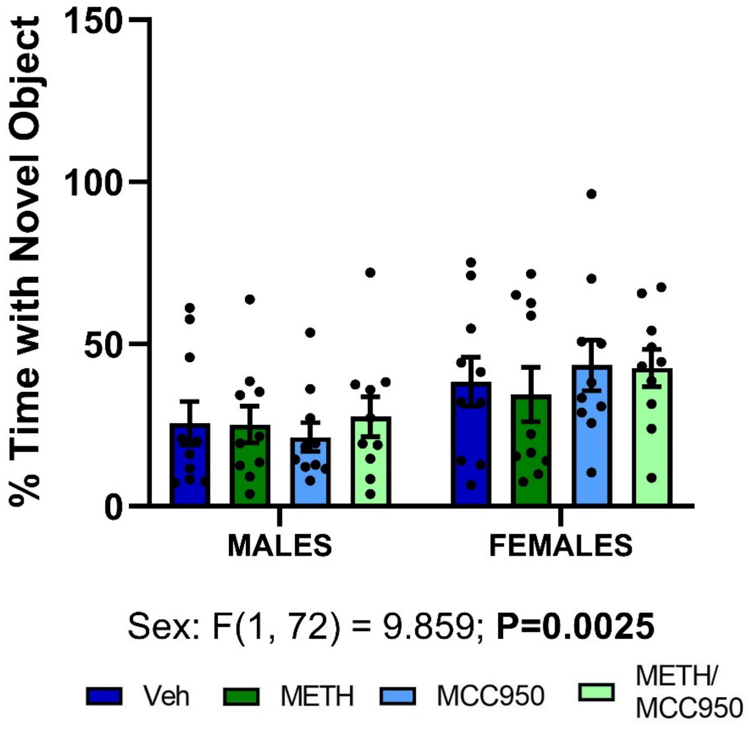

Supplementary Figure S6. Assessment of METH and/or MCC950 administration on recognition memory measured by the Novel Object test. The main effect of sex (P=0.0025) was revealed by a three-way ANOVA. Graphs represent the mean ± SEM (n = 10 per group).

Supplementary Table S1. Evaluation of the estrous cycle in female mice. Percent of female mice in each phase of the estrous cycle.

Mice subjected to OF/NOR (%)				
	Proestrus	Diestrus	Metestrus	Estrus
Veh group	0	33.3	55.6	11.1
METH group	0	50	20	30
MCC950 group	0	66.7	33.3	0
METH/MCC950 group	0	44.4	33.4	22.2
**All female tested mice**	**0**	**47.4**	**36.8**	**15.8**
**Mice subjected to Morris Water Maze (%)**				
	Proestrus	Diestrus	Metestrus	Estrus
Veh group	9.1	27.3	63.6	0
METH group	0	25.0	50.0	25.0
MCC950 group	0	25.0	75.0	0
METH/MCC950 group	0	20.0	80.0	0
**All female tested mice**	**2.4**	**24.4**	**65.9**	**7.3**

Supplementary Table S2 Producers, catalog numbers of primary antibodies, blocking buffers, antibodies diluents, and dilutions of primary and secondary antibodies.

Immunoblotting						
Primary antibody	Producer. catalog number	Dilution of primary antibody	Blocking buffer	Primary antibody diluent	Secondary antibody diluent	Dilution of secondary antibody
anti-ANXA3	Proteintech. 11804-1-AP	1:1000	1x TBST with 5% NFDM	1x TBST with 5% NFDM	1x TBST with 5% NFDM	1:4000
anti-ASC	Cell Signaling. 67824T	1:1000	1x TBST with 5% NFDM	1x TBST with 5% BSA	1x TBST with 5% NFDM	1:4000
anti-AIM2	Affinity. DF3514	1:750	1x TBST with 5% NFDM	1x TBST with 5% NFDM	1x TBST with 5% NFDM	1:4000
anti-CEND1	Proteintech. 13280-1-AP	1:1000	1x TBST with 5% NFDM	1x TBST with 5% NFDM	1x TBST with 5% NFDM	1:4000
anti-DCX	Abcam. ab18723	1:1000	1x TBST with 5% NFDM	1x TBST with 5% NFDM	1x TBST with 5% NFDM	1:4000
anti-FGF12	Proteintech. 13784-1-AP	1:4500	1x TBST with 5% NFDM	1x TBST with 5% NFDM	1x TBST with 5% NFDM	1:4000
anti-HINT1	Proteintech. 10717-1-AP	1:500	1x TBST with 5% NFDM	1x TBST with 5% NFDM	1x TBST with 5% NFDM	1:4000
anti-NeuN	Abcam. ab177487	1:1250	1x TBST with 5% NFDM	1x TBST with 5% NFDM	1x TBST with 5% NFDM	1:5000
anti-NLRP1	Biorbyt. ORB1108082	1:500	1x TBST with 5% NFDM	1x TBST with 5% NFDM	1x TBST with 5% NFDM	1:4000
anti-NLRP3	Abcam. ab270449	1:750	1x TBST with 5% NFDM	1x TBST with 5% NFDM	1x TBST with 5% NFDM	1:4000
anti-NLRC4	Affinity. DF13673	1:1000	1x TBST with 5% NFDM	1x TBST with 5% NFDM	1x TBST with 5% NFDM	1:4000
anti-pro Caspase-1	Abcam, ab179515	1:1000	1x TBST with 5% NFDM	1x TBST with 5% NFDM	1x TBST with 5% NFDM	1:4000
anti-RRBP1	Proteintech. 22015-1-AP	1:4000	1x TBST with 5% NFDM	1x TBST with 5% NFDM	1x TBST with 5% NFDM	1:4000
anti-ZO1	Abcam. ab96587	1:1000	1x TBST with 5% NFDM	1x TBST with 5% NFDM	1x TBST with 5% NFDM	1:4000
anti-occludin	Cell Signaling.	1:1000	1x TBST with 5% NFDM	1x TBST with 5% NFDM	1x TBST with 5% NFDM	1:4000

<table>
<tr><td></td><td>91131S</td><td></td><td></td><td></td><td></td><td></td></tr>
<tr><td>anti-claudin-5</td><td>Sigma Aldrich SAB4502981</td><td>1:1000</td><td>1x TBST with 5% NFDM</td><td>1x TBST with 5% NFDM</td><td>1x TBST with 5% NFDM</td><td>1:4000</td></tr>
<tr><td colspan="7">Secondary antibody</td></tr>
<tr><td colspan="4">Name</td><td colspan="3">Producer. catalog number</td></tr>
<tr><td colspan="4">Peroxidase AffiniPureTM Goat Anti-Rabbit IgG</td><td colspan="3">Jackson ImmunoResearch Laboratories. 111-035-003</td></tr>
<tr><td colspan="7">Immunofluorescence</td></tr>
<tr><td>Primary antibody</td><td>Producer. catalog number</td><td>Dilution of primary antibody</td><td>Blocking buffer</td><td>Primary antibody diluent</td><td>Secondary antibody diluent</td><td>Dilution of secondary antibody</td></tr>
<tr><td>anti-BrdU</td><td>Abcam. ab1893</td><td>1:1000</td><td>5% donkey serum</td><td>Antibody Diluent Reagent Solution Ready-to-use. Invitrogen. 003218</td><td>Antibody Diluent Reagent Solution Ready-to-use. Invitrogen. 003218</td><td>1:2000</td></tr>
<tr><td>anti-NeuN</td><td>Abcam. ab279297</td><td>1:1000</td><td>5% donkey serum</td><td>Antibody Diluent Reagent Solution Ready-to-use. Invitrogen. 003218</td><td>Antibody Diluent Reagent Solution Ready-to-use. Invitrogen. 003218</td><td>1:2000</td></tr>
<tr><td>anti-NG2</td><td>Invitrogen. MA5-24247</td><td>1:1000</td><td>5% donkey serum</td><td>Antibody Diluent Reagent Solution Ready-to-use. Invitrogen. 003218</td><td>Antibody Diluent Reagent Solution Ready-to-use. Invitrogen. 003218</td><td>1:2000</td></tr>
<tr><td colspan="7">Secondary antibody</td></tr>
<tr><td colspan="4">Name</td><td colspan="3">Producer, catalog number</td></tr>
<tr><td colspan="4">Donkey Anti-Sheep IgG H&L (Alexa Fluor® 594</td><td colspan="3">Abcam, ab150180</td></tr>
<tr><td colspan="4">Donkey Anti-Rat IgG H&L (Alexa Fluor® 647) preadsorbed</td><td colspan="3">Abcam, ab150155</td></tr>
<tr><td colspan="4">Donkey Anti-Rabbit IgG H&L (Alexa Fluor® 488) preadsorbed</td><td colspan="3">Abcam, ab150061</td></tr>
</table>

Supplementary Table S3. Statistical source data for the extended data from the ANOVA test.

**Figure 2**				
	Type of test	F (DFn, DFd)<br>P-value for<br>Sex factor	F (DFn, DFd).<br>P-value for<br>METH factor	F (DFn, DFd).<br>P-value for METH x Sex factor
NLRP3	two-way ANOVA	F (1, 19) = 0.9640<br>P=0.3385	F (1, 19) = 3.198<br>P=0.0897	F (1, 19) = 0.9640<br>P=0.3385
ASC	two-way ANOVA	F (1, 19) = 1.103<br>P=0.3068	F (1, 19) = 16.77<br>**P=0.0006**	F (1, 19) = 1.103<br>P=0.3068
Pro-cas1	two-way ANOVA	F (1, 19) = 1.066<br>P=0.3148	F (1, 19) = 2.340<br>P=0.1426	F (1, 19) = 1.066<br>P=0.3148
NLRP1	two-way ANOVA	F (1, 20) = 1.551<br>P=0.2275	F (1, 20) = 0.01687<br>P=0.8980	F (1, 20) = 1.551<br>P=0.2275
NLRC4	two-way ANOVA	F (1, 20) = 3.431<br>P=0.0788	F (1, 20) = 0.8217<br>P=0.3755	F (1, 20) = 3.435<br>P=0.0786
AIM2	two-way ANOVA	F (1, 20) = 0.0015<br>P=0.9696	F (1, 20) = 5.8<br>**P=0.0261**	F (1, 20) = 0.0015<br>P=0.9696

**Figure 3**								
	Type of test	F (DFn. DFd).<br>P-value for<br>Sex factor	F (DFn. DFd).<br>P-value for<br>MCC950 factor	F (DFn. DFd).<br>P-value for<br>METH factor	F (DFn. DFd).<br>P-value for<br>Sex x MCC950 factor	F (DFn. DFd).<br>P-value for<br>Sex x METH factor	F (DFn. DFd).<br>P-value for<br>MCC950 x METH factor	F (DFn. DFd).<br>P-value for<br>Sex x MCC950 x METH factor
ZO-1	three-way ANOVA	F (1, 39) = 3.25<br>P=0.0643	F (1, 39) = 64.26<br>**P<0.0001**	F (1, 39) = 0.0016<br>P=0.9682	F (1, 39) = 4.513<br>**P=0.0400**	F (1, 39) = 0.8194<br>P=0.3709	F (1, 39) = 0.005117<br>P=0.9433	F (1, 39) = 1.267<br>P=0.2671
Cldn-5	three-way ANOVA	F (1, 39) = 21.13<br>**P<0.0001**	F (1, 39) = 2.077<br>P=0.1575	F (1, 39) = 0.05241<br>P=0.8201	F (1, 39) = 17.34<br>**P=0.0002**	F (1, 39) = 1.407<br>P=0.2427	F (1, 39) = 2.937<br>P=0.0945	F (1, 39) = 0.5677<br>P=0.4557
Ocl	three-way ANOVA	F (1, 39) = 4.248<br>**P=0.0460**	F (1, 39) = 2.835<br>P=0.1002	F (1, 39) = 2.452<br>P=0.1255	F (1, 39) = 0.3728<br>P=0.5450	F (1, 39) = 1.604<br>P=0.2129	F (1, 39) = 0.03948<br>P=0.8435	F (1, 39) = 0.03386<br>P=0.8550

**Figure 4**								
	Type of test	F (DFn. DFd).<br>P-value for	F (DFn. DFd).<br>P-value for	F (DFn. DFd).<br>P-value for	F (DFn. DFd).<br>P-value for	F (DFn. DFd).	F (DFn. DFd).	F (DFn. DFd).

		Sex factor	MCC950 factor	METH factor	Sex x MCC950 factor	P-value for Sex x METH factor	P-value for MCC950 x METH factor	P-value for Sex x MCC950 x METH factor
Eotaxin (Serum)	three-way ANOVA	F (1, 24) = 0.3894 P=0.5385	F (1, 24) = 27.72 **P<0.0001**	F (1, 24) = 4.758 P=0.0392	F (1, 24) = 3.828 P=0.0621	F (1, 24) = 1.847 P=0.1868	F (1, 24) = 0.5131 P=0.4807	F (1, 24) = 0.05301 P=0.8199
G-CSF (Serum)	three-way ANOVA	F (1, 24) = 4.281 **P=0.0495**	F (1, 24) = 18.07 **P=0.0003**	F (1, 24) = 12.96 **P=0.0014**	F (1, 24) = 4.866 **P=0.0372**	F (1, 24) = 4.770 **P=0.0390**	F (1, 24) = 6.800 **P=0.0154**	F (1, 24) = 11.40 **P=0.0025**
GM-CSF (Serum)	three-way ANOVA	F (1, 24) = 60.96 **P<0.0001**	F (1, 24) = 4.618 P=0.0549	F (1, 24) = 60.96 **P<0.0001**	F (1, 24) = 4.617 **P=0.0420**	F (1, 24) = 60.96 **P<0.0001**	F (1, 24) = 4.618 P=0.0519	F (1, 24) = 4.617 P=0.0620
IFN-ɣ (Serum)	three-way ANOVA	F (1, 24) = 3.673 P=0.0673	F (1, 24) = 0.5435 P=0.4681	F (1, 24) = 3.358 P=0.0793	F (1, 24) = 0.009387 P=0.9236	F (1, 24) = 2.873 P=0.1030	F (1, 24) = 0.03755 P=0.8480	F (1, 24) = 0.08352 P=0.7751
IL-1α (Serum)	three-way ANOVA	F (1, 24) = 16.75 **P=0.0004**	F (1, 24) = 2.412 P=0.1335	F (1, 24) = 7.044 **P=0.0139**	F (1, 24) = 5.113 **P=0.0331**	F (1, 24) = 7.179 **P=0.0131**	F (1, 24) = 0.7264 P=0.4025	F (1, 24) = 0.6313 P=0.4347
IL-1β (Serum)	three-way ANOVA	F (1, 24) = 0.1484 P=0.7034	F (1, 24) = 59.24 **P<0.0001**	F (1, 24) = 11.24 **P=0.0027**	F (1, 24) = 0.6731 P=0.4201	F (1, 24) = 0.1646 P=0.6885	F (1, 24) = 17.04 **P=0.0004**	F (1, 24) = 0.5413 P=0.4690
IL-2 (Serum)	three-way ANOVA	F (1, 24) = 12.92 **P=0.0015**	F (1, 24) = 0.3930 P=0.5366	F (1, 24) = 9.882 **P=0.0044**	F (1, 24) = 4.442 **P=0.0457**	F (1, 24) = 13.58 **P=0.0012**	F (1, 24) = 0.3549 P=0.5569	F (1, 24) = 3.743 P=0.0649
IL-3 (Serum)	three-way ANOVA	F (1, 24) = 14.64 **P=0.0008**	F (1, 24) = 1.827 P=0.1890	F (1, 24) = 0.7964 P=0.3810	F (1, 24) = 0.8045 P=0.3786	F (1, 24) = 0.09881 P=0.7560	F (1, 24) = 0.01112 P=0.9169	F (1, 24) = 0.1324 P=0.7191
IL-4 (Serum)	three-way ANOVA	F (1, 24) = 1.240 P=0.2765	F (1, 24) = 14.90 **P=0.0007**	F (1, 24) = 5.634 **P=0.0260**	F (1, 24) = 8.170 **P=0.0087**	F (1, 24) = 4.548 **P=0.0434**	F (1, 24) = 0.3630 P=0.5525	F (1, 24) = 4.772 **P=0.0389**
IL-5 (Serum)	three-way ANOVA	F (1, 24) = 1.396 P=0.2489	F (1, 24) = 0.3467 P=0.5615	F (1, 24) = 0.01471 P=0.9045	F (1, 24) = 0.5388 P=0.4700	F (1, 24) = 0.1944 P=0.6632	F (1, 24) = 0.4104 P=0.5278	F (1, 24) = 0.1362 P=0.7153

IL-6 (Serum)	three-way ANOVA	F (1, 24) = 25.22 **P<0.0001**	F (1, 24) = 16.57 **P=0.0004**	F (1, 24) = 26.24 **P<0.0001**	F (1, 24) = 18.87 **P=0.0002**	F (1, 24) = 18.34 **P=0.0003**	F (1, 24) = 29.19 **P<0.0001**	F (1, 24) = 25.85 **P<0.0001**
IL-9 (Serum)	three-way ANOVA	F (1, 24) = 1.537 P=0.2271	F (1, 24) = 1.859 P=0.1854	F (1, 24) = 0.01001 P=0.9211	F (1, 24) = 12.34 **P=0.0018**	F (1, 24) = 0.07615 P=0.7849	F (1, 24) = 0.006412 P=0.9368	F (1, 24) = 0.01706 P=0.8972
IL-10 (Serum)	three-way ANOVA	F (1, 24) = 24.65 **P<0.0001**	F (1, 24) = 47.00 **P<0.0001**	F (1, 24) = 1.560 P=0.2237	F (1, 24) = 36.90 **P<0.0001**	F (1, 24) = 11.06 **P=0.0028**	F (1, 24) = 0.2012 P=0.6578	F (1, 24) = 7.770 **P=0.0102**
IL-12(p40) (Serum)	three-way ANOVA	F (1, 24) = 0.2458 P=0.6246	F (1, 24) = 0.8969 P=0.3531	F (1, 24) = 5.544 **P=0.0271**	F (1, 24) = 9.926 **P=0.0043**	F (1, 24) = 0.6833 P=0.4166	F (1, 24) = 0.5746 P=0.4558	F (1, 24) = 0.01862 P=0.8926
IL-12(p70) (Serum)	three-way ANOVA	F (1, 24) = 8.836 **P=0.0066**	F (1, 24) = 114.0 **P<0.0001**	F (1, 24) = 22.60 **P<0.0001**	F (1, 24) = 16.31 **P=0.0005**	F (1, 24) = 44.25 **P<0.0001**	F (1, 24) = 2.360 P=0.1376	F (1, 24) = 15.31 **P=0.0007**
IL-13 (Serum)	three-way ANOVA	F (1, 24) = 18.44 **P=0.0002**	F (1, 24) = 71.81 **P<0.0001**	F (1, 24) = 30.17 **P<0.0001**	F (1, 24) = 47.90 **P<0.0001**	F (1, 24) = 1.027 P=0.3210	F (1, 24) = 19.23 **P=0.0002**	F (1, 24) = 0.3112 P=0.5821
IL-17A (Serum)	three-way ANOVA	F (1, 24) = 34.18 **P<0.0001**	F (1, 24) = 36.11 **P<0.0001**	F (1, 24) = 5.230 **P=0.0313**	F (1, 24) = 51.17 **P<0.0001**	F (1, 24) = 0.05288 P=0.8201	F (1, 24) = 0.02041 P=0.8876	F (1, 24) = 0.4426 P=0.5122
KC (CXCL1) (Serum)	three-way ANOVA	F (1, 24) = 44.50 **P<0.0001**	F (1, 24) = 78.63 **P<0.0001**	F (1, 24) = 59.69 **P<0.0001**	F (1, 24) = 29.41 **P<0.0001**	F (1, 24) = 53.32 **P<0.0001**	F (1, 24) = 79.51 **P<0.0001**	F (1, 24) = 83.51 **P<0.0001**
MCP-1 (Serum)	three-way ANOVA	F (1, 24) = 1.081 P=0.3088	F (1, 24) = 27.14 **P<0.0001**	F (1, 24) = 15.14 **P=0.0007**	F (1, 24) = 19.67 **P=0.0002**	F (1, 24) = 3.289 P=0.0823	F (1, 24) = 3.632 P=0.0687	F (1, 24) = 0.9017 P=0.3518
MIP-1α (Serum)	three-way ANOVA	F (1, 24) = 2.396 P=0.1348	F (1, 24) = 1.499 P=0.2327	F (1, 24) = 0.1308 P=0.7208	F (1, 24) = 0.5702 P=0.4575	F (1, 24) = 4.220 **P=0.0510**	F (1, 24) = 0.001053 P=0.9744	F (1, 24) = 0.2783 P=0.6026

MIP-1β (Serum)	three-way ANOVA	F (1, 24) = 0.9040 P=0.3512	F (1, 24) = 41.25 **P<0.0001**	F (1, 24) = 25.73 **P<0.0001**	F (1, 24) = 1.501 P=0.2324	F (1, 24) = 2.243 P=0.1473	F (1, 24) = 22.88 **P<0.0001**	F (1, 24) = 0.006857 P=0.9347
RANTES (Serum)	three-way ANOVA	F (1, 24) = 6.966 **P=0.0144**	F (1, 24) = 5.808 **P=0.0240**	F (1, 24) = 0.7447 P=0.3967	F (1, 24) = 13.92 **P=0.0010**	F (1, 24) = 6.651 **P=0.0165**	F (1, 24) = 20.34 **P=0.0001**	F (1, 24) = 12.43 **P=0.0017**
TNF-α (Serum)	three-way ANOVA	F (1, 24) = 20.72 **P=0.0001**	F (1, 24) = 86.67 **P<0.0001**	F (1, 24) = 21.20 **P=0.0001**	F (1, 24) = 49.81 **P<0.0001**	F (1, 24) = 0.6331 P=0.4340	F (1, 24) = 24.93 **P<0.0001**	F (1, 24) = 0.2599 P=0.6149
Eotaxin (Hippocampus)	three-way ANOVA	F (1, 24) = 266.4 **P<0.0001**	F (1, 24) = 6.079 **P=0.0212**	F (1, 24) = 257.8 **P<0.0001**	F (1, 24) = 0.04224 P=0.8389	F (1, 24) = 320.1 **P<0.0001**	F (1, 24) = 0.6383 P=0.4322	F (1, 24) = 9.322 **P=0.0055**
G-CSF (Hippocampus)	three-way ANOVA	F (1, 24) = 33.35 **P<0.0001**	F (1, 24) = 3.000 P=0.0961	F (1, 24) = 15.04 **P=0.0007**	F (1, 24) = 7.166 **P=0.0132**	F (1, 24) = 7.816 **P=0.0100**	F (1, 24) = 2.504 P=0.1266	F (1, 24) = 0.08516 P=0.7729
GM-CSF (Hippocampus)	three-way ANOVA	F (1, 24) = 58.64 **P<0.0001**	F (1, 24) = 0.6108 P=0.4421	F (1, 24) = 16.34 **P=0.0005**	F (1, 24) = 3.872 P=0.0608	F (1, 24) = 25.60 **P<0.0001**	F (1, 24) = 7.934 **P=0.0096**	F (1, 24) = 0.2106 P=0.6504
IFN-ɣ (Hippocampus)	three-way ANOVA	F (1, 24) = 17.98 **P=0.0003**	F (1, 24) = 0.09576 P=0.7596	F (1, 24) = 0.08220 P=0.7768	F (1, 24) = 0.1065 P=0.7470	F (1, 24) = 4.176 P=0.0521	F (1, 24) = 1.352 P=0.2563	F (1, 24) = 1.418 P=0.2453
IL-1α (Hippocampus)	three-way ANOVA	F (1, 24) = 84.72 **P<0.0001**	F (1, 24) = 3.185 P=0.0870	F (1, 24) = 68.12 **P<0.0001**	F (1, 24) = 0.1045 P=0.7493	F (1, 24) = 41.84 **P<0.0001**	F (1, 24) = 1.677 P=0.2076	F (1, 24) = 0.0002026 P=0.9888
IL-1β (Hippocampus)	three-way ANOVA	F (1, 24) = 301.1 **P<0.0001**	F (1, 24) = 2.271 P=0.1449	F (1, 24) = 270.0 **P<0.0001**	F (1, 24) = 32.44 **P<0.0001**	F (1, 24) = 101.2 **P<0.0001**	F (1, 24) = 0.7422 P=0.3975	F (1, 24) = 52.63 **P<0.0001**
IL-2 (Hippocampus)	three-way ANOVA	F (1, 24) = 176.0 **P<0.0001**	F (1, 24) = 3.944 P=0.0586	F (1, 24) = 224.0 **P<0.0001**	F (1, 24) = 4.648 **P=0.0413**	F (1, 24) = 218.9 **P<0.0001**	F (1, 24) = 16.25 **P=0.0005**	F (1, 24) = 1.206 P=0.2831

IL-3 (Hippocampus)	three-way ANOVA	F (1, 24) = 57.01 **P<0.0001**	F (1, 24) = 4.602 **P=0.0423**	F (1, 24) = 44.34 **P<0.0001**	F (1, 24) = 6.372 **P=0.0186**	F (1, 24) = 77.72 **P<0.0001**	F (1, 24) = 0.1960 P=0.6619	F (1, 24) = 2.264 P=0.1455
IL-4 (Hippocampus)	three-way ANOVA	F (1, 24) = 103.2 **P<0.0001**	F (1, 24) = 2.426 P=0.1325	F (1, 24) = 67.50 **P<0.0001**	F (1, 24) = 9.423 **P=0.0053**	F (1, 24) = 26.95 **P<0.0001**	F (1, 24) = 0.1966 P=0.6615	F (1, 24) = 7.051 **P=0.0139**
IL-5 (Hippocampus)	three-way ANOVA	F (1, 24) = 3.685 P=0.0669	F (1, 24) = 1.117 P=0.3011	F (1, 24) = 0.003572 P=0.9528	F (1, 24) = 8.981 **P=0.0063**	F (1, 24) = 8.877 **P=0.0065**	F (1, 24) = 15.91 **P=0.0005**	F (1, 24) = 0.3272 P=0.5726
IL-6 (Hippocampus)	three-way ANOVA	F (1, 24) = 35.66 **P<0.0001**	F (1, 24) = 1.929 P=0.1777	F (1, 24) = 11.36 **P=0.0025**	F (1, 24) = 2.707 P=0.1129	F (1, 24) = 24.05 **P<0.0001**	F (1, 24) = 0.01700 P=0.8974	F (1, 24) = 0.07760 P=0.7830
IL-9 (Hippocampus)	three-way ANOVA	F (1, 24) = 153.9 **P<0.0001**	F (1, 24) = 17.08 **P=0.0004**	F (1, 24) = 65.00 **P<0.0001**	F (1, 24) = 4.011 P=0.0566	F (1, 24) = 192.2 **P<0.0001**	F (1, 24) = 6.492 **P=0.0177**	F (1, 24) = 0.1852 P=0.6708
IL-10 (Hippocampus)	three-way ANOVA	F (1, 24) = 164.3 **P<0.0001**	F (1, 24) = 2.223 P=0.1490	F (1, 24) = 96.15 **P<0.0001**	F (1, 24) = 3.204 P=0.0861	F (1, 24) = 229.6 **P<0.0001**	F (1, 24) = 9.054 **P=0.0061**	F (1, 24) = 0.1172 P=0.7351
IL-12(p40) (Hippocampus)	three-way ANOVA	F (1, 24) = 114.6 **P<0.0001**	F (1, 24) = 0.1115 P=0.7414	F (1, 24) = 61.18 **P<0.0001**	F (1, 24) = 0.1401 P=0.7115	F (1, 24) = 63.07 **P<0.0001**	F (1, 24) = 0.05601 P=0.8149	F (1, 24) = 16.39 **P=0.0005**
IL-12(p70) (Hippocampus)	three-way ANOVA	F (1, 24) = 40.12 **P<0.0001**	F (1, 24) = 1.693 P=0.2056	F (1, 24) = 152.0 **P<0.0001**	F (1, 24) = 0.6479 P=0.4288	F (1, 24) = 56.69 **P<0.0001**	F (1, 24) = 57.87 **P<0.0001**	F (1, 24) = 98.23 **P<0.0001**
IL-13 (Hippocampus)	three-way ANOVA	F (1, 24) = 102.0 **P<0.0001**	F (1, 24) = 18.78 **P=0.0002**	F (1, 24) = 318.0 **P<0.0001**	F (1, 24) = 29.22 **P<0.0001**	F (1, 24) = 348.6 **P<0.0001**	F (1, 24) = 2.643 P=0.1170	F (1, 24) = 22.74 **P<0.0001**
IL-17A (Hippocampus)	three-way ANOVA	F (1, 24) = 85.98 **P<0.0001**	F (1, 24) = 11.89 **P=0.0021**	F (1, 24) = 44.10 **P<0.0001**	F (1, 24) = 9.682 **P=0.0048**	F (1, 24) = 83.19 **P<0.0001**	F (1, 24) = 21.54 **P=0.0001**	F (1, 24) = 2.774 P=0.1088

KC (CXCL1) (Hippocampus)	three-way ANOVA	F (1, 24) = 173.7 **P<0.0001**	F (1, 24) = 0.2981 P=0.5901	F (1, 24) = 204.8 **P<0.0001**	F (1, 24) = 0.7007 P=0.4108	F (1, 24) = 106.6 **P<0.0001**	F (1, 24) = 5.262 **P=0.0308**	F (1, 24) = 3.526 P=0.0726
MCP-1 (Hippocampus)	three-way ANOVA	F (1, 24) = 593.7 **P<0.0001**	F (1, 24) = 4.050 P=0.0555	F (1, 24) = 371.2 **P<0.0001**	F (1, 24) = 1.976 P=0.1727	F (1, 24) = 532.2 **P<0.0001**	F (1, 24) = 0.05506 P=0.8165	F (1, 24) = 44.10 **P<0.0001**
MIP-1α (Hippocampus)	three-way ANOVA	F (1, 24) = 47.47 **P<0.0001**	F (1, 24) = 4.220 P=0.0510	F (1, 24) = 5.359 **P=0.0295**	F (1, 24) = 0.2579 P=0.6162	F (1, 24) = 4.219 P=0.0510	F (1, 24) = 1.006 P=0.3258	F (1, 24) = 4.143 P=0.0530
MIP-1β (Hippocampus)	three-way ANOVA	F (1, 24) = 642.4 **P<0.0001**	F (1, 24) = 35.11 **P<0.0001**	F (1, 24) = 237.7 **P<0.0001**	F (1, 24) = 29.15 **P<0.0001**	F (1, 24) = 731.4 **P<0.0001**	F (1, 24) = 0.8303 P=0.3713	F (1, 24) = 0.9179 P=0.3476
RANTES (Hippocampus)	three-way ANOVA	F (1, 24) = 414.4 **P<0.0001**	F (1, 24) = 2.493 P=0.1275	F (1, 24) = 470.3 **P<0.0001**	F (1, 24) = 28.22 **P<0.0001**	F (1, 24) = 322.3 **P<0.0001**	F (1, 24) = 7.478 **P=0.0116**	F (1, 24) = 0.7212 P=0.4041
TNF-α (Hippocampus)	three-way ANOVA	F (1, 24) = 402.9 **P<0.0001**	F (1, 24) = 29.76 **P<0.0001**	F (1, 24) = 33.95 **P<0.0001**	F (1, 24) = 0.1194 P=0.7327	F (1, 24) = 107.3 **P<0.0001**	F (1, 24) = 418.5 **P<0.0001**	F (1, 24) = 333.7 **P<0.0001**

Figure 5

	Type of test	F (DFn. DFd). P-value for Sex factor	F (DFn. DFd). P-value for Time factor	F (DFn. DFd). P-value for METH factor	F (DFn. DFd). P-value for MCC950 factor	F (DFn. DFd). P-value for Sex x Time factor	F (DFn. DFd). P-value for Sex x METH factor	F (DFn. DFd). P-value for Time x METH factor	F (DFn. DFd). P-value for Sex x MCC950 factor	F (DFn. DFd). P-value for Time x MCC950 factor	F (DFn. DFd). P-value for METH x MCC950 factor	F (DFn. DFd). P-value for Sex x Time x METH factor	F (DFn. DFd). P-value for Sex x Time x MCC950 factor	F (DFn. DFd). P-value for Sexr x METH x MCC950 factor	F (DFn. DFd). P-value for Time x METH x MCC950 factor	F (DFn. DFd). P-value for Sex x Time x METH x MCC950 factor
BrdU	four-way ANOVA	F (1, 96) = 5.3831 **P = 0.022451**	F (5. 96) = 3.8817 **P = 0.00301**	F (1, 96) = 8.8119 **P = 0.003779**	F (1, 96) = 14.6654 **P = 0.00023**	F (5. 96) = 6.6714 **P = 0.000023**	F (1, 96) = 0.0015 P = 0.969577	F (5. 96) = 1.9892 P = 0.087089	F (1, 96) = 0.4770 P = 0.491448	F (5. 96) = 1.5702 P = 0.175826	F (1, 96) = 35.6839 **P = 0.000001**	F (5. 96) = 2.7465 **P = 0.023081**	F (5. 96) = 1.9695 P = 0.090068	F (1, 96) = 5.1105 **P = 0.026040**	F (5. 96) = 1.5837 **P = 0.171983**	F (5. 96) = 5.1650 **P = 0.000307**

	Type of test	F (DFn. DFd). P-value for Sex factor	F (DFn. DFd). P-value for MCC950 factor	F (DFn. DFd). P-value for METH factor	F (DFn. DFd). P-value for Sex x MCC950 factor	F (DFn. DFd). P-value for Sex x METH factor	F (DFn. DFd). P-value for MCC950 x METH factor	F (DFn. DFd). P-value for Sex x MCC950 x METH factor

BrdU (D5)	three-way ANOVA	F (1, 16) = 12.39 **P=0.0028**	F (1, 16) = 7.375 **P=0.0153**	F (1, 16) = 2.111 P=0.1656	F (1, 16) = 2.456 P=0.1366	F (1, 16) = 4.284 P=0.0550	F (1, 16) = 1.930 P=0.1838	F (1, 16) = 11.59 **P=0.0036**
BrdU (D9)	three-way ANOVA	F (1, 16) = 26.23 **P=0.0001**	F (1, 16) = 0.1108 P=0.7435	F (1, 16) = 16.99 **P=0.0008**	F (1, 16) = 0.01338 P=0.9093	F (1, 16) = 0.03152 P=0.8613	F (1, 16) = 7.085 **P=0.0171**	F (1, 16) = 1.344 P=0.2634
BrdU (D13)	three-way ANOVA	F (1, 16) = 7.797 **P=0.0130**	F (1, 16) = 6.721 **P=0.0196**	F (1, 16) = 9.536 **P=0.0071**	F (1, 16) = 5.704 **P=0.0296**	F (1, 16) = 3.684 P=0.0729	F (1, 16) = 1.256 P=0.2789	F (1, 16) = 2.691 P=0.1205
BrdU (D17)	three-way ANOVA	F (1, 16) = 0.1261 P=0.7271	F (1, 16) = 0.2363 P=0.6335	F (1, 16) = 3.597 P=0.0761	F (1, 16) = 3.773 P=0.0699	F (1, 16) = 0.3077 P=0.5867	F (1, 16) = 11.85 **P=0.0033**	F (1, 16) = 6.468 **P=0.0217**
BrdU (D21)	three-way ANOVA	F (1, 16) = 6.980 **P=0.0178**	F (1, 16) = 11.89 **P=0.0033**	F (1, 16) = 3.130 P=0.0959	F (1, 16) = 0.006007 P=0.9392	F (1, 16) = 10.03 **P=0.0060**	F (1, 16) = 28.45 **P<0.0001**	F (1, 16) = 2.202 P=0.1573
BrdU (D25)	three-way ANOVA	F (1, 16) = 2.016 P=0.1748	F (1, 16) = 1.509 P=0.2370	F (1, 16) = 0.02095 P=0.8867	F (1, 16) = 0.05074 P=0.8246	F (1, 16) = 0.4834 P=0.4969	F (1, 16) = 5.217 **P=0.0364**	F (1, 16) = 1.802 P=0.1983
**Figure 6**								
	Type of test	F (DFn. DFd). P-value for Sex factor	F (DFn. DFd). P-value for MCC950 factor	F (DFn. DFd). P-value for METH factor	F (DFn. DFd). P-value for Sex x MCC950 factor	F (DFn. DFd). P-value for Sex x METH factor	F (DFn. DFd). P-value for MCC950 x METH factor	F (DFn. DFd). P-value for Sex x MCC950 x METH factor
DCX	three-way ANOVA	F (1, 16) = 1.933 P=0.1834	F (1, 16) = 7.302 **P=0.0157**	F (1, 16) = 3.515 P=0.0792	F (1, 16) = 6.303 **P=0.0232**	F (1, 16) = 2.489 P=0.1342	F (1, 16) = 0.8265 P=0.3768	F (1, 16) = 0.1235 P=0.7299
NeuN	three-way ANOVA	F (1, 16) = 5.106 **P=0.0381**	F (1, 16) = 0.09273 P=0.7647	F (1, 16) = 0.2482 P=0.6251	F (1, 16) = 0.01567 P=0.9019	F (1, 16) = 0.6222 P=0.4418	F (1, 16) = 0.006340 P=0.9375	F (1, 16) = 1.646 P=0.2178

DCX/NeuN	three-way ANOVA	F (1, 16) = 0.02272 P=0.8821	F (1, 16) = 9.924 **P=0.0062**	F (1, 16) = 3.831 P=0.0680	F (1, 16) = 6.071 **P=0.0254**	F (1, 16) = 2.957 P=0.1048	F (1, 16) = 0.4094 P=0.5313	F (1, 16) = 0.8501 P=0.3702

Figure 7

	Type of test	F (DFn. DFd). P-value for Sex factor	F (DFn. DFd). P-value for Time factor	F (DFn. DFd). P-value for METH factor	F (DFn. DFd). P-value for MCC950 factor	F (DFn. DFd). P-value for Sex x Time factor	F (DFn. DFd). P-value for Sexr x METH factor	F (DFn. DFd). P-value for Time x METH factor	F (DFn. DFd). P-value for Sex x MCC950 factor	F (DFn. DFd). P-value for Time x MCC950 factor	F (DFn. DFd). P-value for METH x MCC950 factor	F (DFn. DFd). P-value for Sex x Time x METH factor	F (DFn. DFd). P-value for Sex x Time x MCC950 factor	F (DFn. DFd). P-value for Sex x METH x MCC950 factor	F (DFn. DFd). P-value for Time x METH x MCC950 factor	F (DFn. DFd). P-value for Sex x Time x METH x MCC950 factor
NG2	four-way ANOVA	F (1, 96) = 22.749 **P = 0.000007**	F (5. 96) = 4.209 **P = 0.001675**	F (1, 96) = 123.708 **P = 0.000001**	MCC950 factor F (1, 96) = 143.008 **P = 0.000001**	F (5. 96) = 6.285 **P = 0.000044**	F (1, 96) = 1.049 P = 0.308369	F (5. 96) = 2.967 **P = 0.015567**	F (1, 96) = 0.818 P = 0.368156	F (5. 96) = 4.182 **P = 0.001759**	F (1, 96) = 90.734 **P = 0.000001**	F (5. 96) = 5.897 **P = 0.000085**	F (5. 96) = 1.327 P = 0.259207	F (1, 96) = 0.953 P = 0.331318	F (5. 96) = 8.943 **P = 0.000001**	F (5. 96) = 12.947 **P = 0.000001**

	Type of test	F (DFn. DFd). P-value for Sex factor	F (DFn. DFd). P-value for MCC950 factor	F (DFn. DFd). P-value for METH factor	F (DFn. DFd). P-value for Sex x MCC950 factor	F (DFn. DFd). P-value for Sex x METH factor	F (DFn. DFd). P-value for MCC950 x METH factor	F (DFn. DFd). P-value for Sex x MCC950 x METH factor
NG2 (D5)	three-way ANOVA	F (1, 16) = 9.995 **P=0.0060**	F (1, 16) = 3.527 P=0.0787	F (1, 16) = 7.031 **P=0.0174**	F (1, 16) = 0.02154 P=0.8852	F (1, 16) = 0.3121 P=0.5841	F (1, 16) = 10.54 **P=0.0051**	F (1, 16) = 0.9681 P=0.3398
NG2 (D9)	three-way ANOVA	F (1, 16) = 2.976 P=0.1038	F (1, 16) = 14.99 **P=0.0014**	F (1, 16) = 16.63 **P=0.0009**	F (1, 16) = 0.02292 P=0.8816	F (1, 16) = 0.07205 P=0.7918	F (1, 16) = 0.8097 P=0.3816	F (1, 16) = 6.962 **P=0.0179**
NG2 (D13)	three-way ANOVA	F (1, 16) = 14.66 **P=0.0015**	F (1, 16) = 11.19 **P=0.0041**	F (1, 16) = 6.675 **P=0.0200**	F (1, 16) = 2.996 P=0.1027	F (1, 16) = 4.224 P=0.0566	F (1, 16) = 132.6 **P<0.0001**	F (1, 16) = 68.38 **P<0.0001**
NG2 (D17)	three-way ANOVA	F (1, 16) = 1.979 P=0.1786	F (1, 16) = 293.5 **P<0.0001**	F (1, 16) = 307.4 **P<0.0001**	F (1, 16) = 4.634 **P=0.0469**	F (1, 16) = 146.5 **P<0.0001**	F (1, 16) = 147.8 **P<0.0001**	F (1, 16) = 15.91 **P=0.0011**
NG2 (D21)	three-way ANOVA	F (1, 16) = 11.53 **P=0.0037**	F (1, 16) = 30.79 **P<0.0001**	F (1, 16) = 31.48 **P<0.0001**	F (1, 16) = 0.3819 P=0.5453	F (1, 16) = 4.516 **P=0.0495**	F (1, 16) = 3.044 P=0.1002	F (1, 16) = 3.703 P=0.0723

NG2 (D25)	three-way ANOVA	F (1, 16) = 6.582 **P=0.0207**	F (1, 16) = 69.47 **P<0.0001**	F (1, 16) = 24.49 **P=0.0001**	F (1, 16) = 5.747 **P=0.0291**	F (1, 16) = 3.418 P=0.0831	F (1, 16) = 6.635 **P=0.0203**	F (1, 16) = 5.068 **P=0.0388**

Figure 8

	Type of test	F (DFn, DFd) P-value for METH factor	F (DFn. DFd). P-value for MCC950 factor	F (DFn. DFd). P-value for METH x MCC950 factor
CEND1 Proteomic data (MS)	Robust two-way ANOVA	F(1, ∞) = 0.5742; P = 0.4486	F(1, ∞) = 11.0340; **P = 0.0009**	$\chi^2(1)$ = 5.7610; P = **0.0164**
FGF12 Proteomic data (MS)	Robust two-way ANOVA	F(1, ∞) = 0.5119; P = 0.4743	F(1, ∞) = 4.3073; P = **0.0379**	$\chi^2(1)$ = 1.9424; P = 0.1634
CEND1 Western blot data	two-way ANOVA	F (1, 8) = 0.5008; P=0.50	F (1, 8) = 1.346; P=0.28	F (1, 8) = 3.390; P=0.10
FGF12 Western blot data	two-way ANOVA	F (1, 8) = 0,3386; P=0,58	F (1, 8) = 11.72; **P=0.009**	F(1, 8) = 5.32; **P=0.05**
HINT1 Proteomic data (MS)	Robust two-way ANOVA	F(1, ∞) = 2.9678; P = 0.0849	F(1, ∞) = 25.2170; P = **5.1×10^{-7}**	$\chi^2(1)$ = 7.3987; P = **0.0065**
ANXA3 Proteomic data (MS)	Robust two-way ANOVA	F(1, ∞) = 1.1950; P = 0.2743	F(1, ∞) = 53.3455; P = **2.8×10^{-13}**	$\chi^2(1)$ = 307.3003; P < **2.2×10^{-16}**
RRBP1 Proteomic data (MS)	Robust two-way ANOVA	F(1, ∞) = 0.1968; P = 0.6573	F(1, ∞) = 39.3483; P = **3.5×10^{-10}**	$\chi^2(1)$ = 0.0360; P = 0.8495
HINT1 Western blot data	two-way ANOVA	F (1, 8) = 0.1124; P=0.75	F (1, 8) = 22.50; **P=0.001**	F (1, 8) = 1.320; P=0.28
ANXA3 Western blot data	two-way ANOVA	F (1, 8) = 2.180; P=0.18	F (1, 8) = 0.3739; P=0.56	F (1, 8) = 33.54; **P<0.001**
RRBP1 Western blot data	two-way ANOVA	F (1, 8) = 7.796; **P=0.02**	F (1, 8) = 20.26; **P=0.002**	F (1, 8) = 0.003135; P=0.96

Figure 9

	Type of test	F (DFn. DFd). P-value for Sex factor	F (DFn. DFd). P-value for MCC950 factor	F (DFn. DFd). P-value for METH factor	F (DFn. DFd). P-value for Sex x MCC950 factor	F (DFn. DFd). P-value for Sex x METH factor	F (DFn. DFd). P-value for MCC950 x METH factor	F (DFn. DFd). P-value for Sex x MCC950 x METH factor
Time spent in the center	three-way ANOVA	F (1, 72) = 3.863 P=0.0532	F (1, 72) = 29.1 **P<0.0001**	F (1, 72) = 13.36 **P=0.0005**	F (1, 72) = 4.748 **P=0.0326**	F (1, 72) = 1.122 P=0.2931	F (1, 72) = 0.2620 P=0.6103	F (1, 72) = 1.620 P=0.2073

Rearing	three-way ANOVA	F (1, 72) = 0.04549 P=0.8317	F (1, 72) = 16.67 **P=0.0001**	F (1, 72) = 1.485 P=0.2269	F (1, 72) = 0.5803 P=0.4487	F (1, 72) = 2.808 P=0.0981	F (1, 72) = 0.4494 P=0.5048	F (1, 72) = 0.1569 P=0.6932
Grooming	three-way ANOVA	F (1, 72) = 0.06718 P=0.7962	F (1, 72) = 18.51 **P<0.0001**	F (1, 72) = 2.583 P=0.1124	F (1, 72) = 1.185 P=0.2799	F (1, 72) = 4.969 **P=0.0289**	F (1, 72) = 0.1317 P=0.7178	F (1, 72) = 2.927 P=0.0914

Figure 10

	Type of test	F (DFn. DFd). P-value for Sex factor	F (DFn. DFd). P-value for MCC950 factor	F (DFn. DFd). P-value for METH factor	F (DFn. DFd). P-value for Sex x MCC950 factor	F (DFn. DFd). P-value for Sex x METH factor	F (DFn. DFd). P-value for MCC950 x METH factor	F (DFn. DFd). P-value for Sex x MCC950 x METH factor
Velocity - learning phase	three-way ANOVA	F (1, 72) = 14.19 **P=0.0003**	F (1, 72) = 0.1797 P=0.6729	F (1, 72) = 4.086 **P=0.0470**	F (1, 72) = 2.334 P=0.1310	F (1, 72) = 0.2526 P=0.6168	F (1, 72) = 0.3536 P=0.5539	F (1, 72) = 4.414 **P=0.0391**
Escape latency - trial probe	three-way ANOVA	F (1, 72) = 3.880 **P=0.0527**	F (1, 72) = 0.1573 P=0.6929	F (1, 72) = 0.1253 P=0.7244	F (1, 72) = 0.04538 P=0.8319	F (1, 72) = 0.4296 P=0.5143	F (1, 72) = 1.823 P=0.1812	F (1, 72) = 2.537 P=0.1156
Time spent in target quadrant - trial probe	three-way ANOVA	F (1, 61) = 2.383 P=0.1279	F (1, 61) = 130.8 **P<0.0001**	F (1, 61) = 8.959 **P=0.0040**	F (1, 61) = 1.014 P=0.3178	F (1, 61) = 0.0009451 P=0.9756	F (1, 61) = 4.065 **P=0.0482**	F (1, 61) = 6.505 **P=0.0133**

	Type of test	F (DFn. DFd). P-value for Sex factor	F (DFn. DFd). P-value for Time factor	F (DFn. DFd). P-value for METH factor	F (DFn. DFd). P-value for MCC950 factor	F (DFn. DFd). P-value for Sex x Time factor	F (DFn. DFd). P-value for Sexr x METH factor	F (DFn. DFd). P-value for Time x METH factor	F (DFn. DFd). P-value for Sex x MCC950 factor	F (DFn. DFd). P-value for Time x MCC950 factor	F (DFn. DFd). P-value for METH x MCC950 factor	F (DFn. DFd). P-value for Sex x Time x METH factor	F (DFn. DFd). P-value for Sex x Time x MCC950 factor	F (DFn. DFd). P-value for Sex x METH x MCC950 factor	F (DFn. DFd). P-value for Time x METH x MCC950 factor	F (DFn. DFd). P-value for Sex x Time x METH x MCC950 factor
Escape latency - learning phase	four-way ANOVA	F(1, 1441) = 6.195 **P = 0.0129**	F(4, 1441) = 25.294 **P = 0.00001**	F(1, 1441) = 0.304 P = 0.581191	F(1, 1441) = 83.934 **P = 0.00001**	F(4, 1441) = 2.162 P = 0.071135	F(1, 1441) = 11.297 P = **0.000797**	F(4, 1441) = 2.006 P = 0.091348	F(1, 1441) = 8.390 P = **0.00383**	F(4, 1441) = 14.742 **P = 0.00001**	F(1, 1441) = 31.694 **P = 0.00001**	F(4, 1441) = 0.650 P = 0.626831	F(4, 1441) = 1.422 P = 0.224353	F(1, 1441) = 4.480 P = **0.03445**	F(4, 1441) = 0.517 P = 0.723076	F(4, 1441) = 1.123 P = 0.343824

Supplementary

Supplementary Figure S2

	Type of test	F (DFn. DFd). P-value for Sex factor	F (DFn. DFd). P-value for MCC950 factor	F (DFn. DFd). P-value for METH factor	F (DFn. DFd). P-value for Sex x MCC950 factor	F (DFn. DFd). P-value for Sex x METH factor	F (DFn. DFd). P-value for MCC950 x METH factor	F (DFn. DFd). P-value for Sex x MCC950 x METH factor
**AST**	three-way ANOVA	F (1, 40) = 1.984 P=0.1667	F (1, 40) = 8.463 **P=0.0059**	F (1, 40) = 0.3998 P=0.5308	F (1, 40) = 7.891 **P=0.0077**	F (1, 40) = 0.1148 P=0.7365	F (1, 40) = 2.538 P=0.1190	F (1, 40) = 0.8086 P=0,3739
**ALT**	three-way ANOVA	F (1, 40) = 1,554 P=0.2198	F (1, 40) = 6.856 **P=0.0124**	F (1, 40) = 1.944 P=0.1709	F (1, 40) = 1.220 P=0.2760	F (1, 40) = 0.5812 P=0.4503	F (1, 40) = 0.5120 P=0.4784	F (1, 40) = 0.2697 P=0.6064

Supplementary Figure S5

	Type of test	F (DFn. DFd). P-value for Sex factor	F (DFn. DFd). P-value for MCC950 factor	F (DFn. DFd). P-value for METH factor	F (DFn. DFd). P-value for Sex x MCC950 factor	F (DFn. DFd). P-value for Sex x METH factor	F (DFn. DFd). P-value for MCC950 x METH factor	F (DFn. DFd). P-value for Sex x MCC950 x METH factor
Velocity	three-way ANOVA	F (1, 72) = 2.977 P=0.0887	F (1, 72) = 2.864 P=0.0949	F (1, 72) = 1.902 P=0.1721	F (1, 72) = 0.7295 P=0.3959	F (1, 72) = 0.09325 P=0.7610	F (1, 72) = 0.04105 P=0.8400	F (1, 72) = 0.9741 P=0.3270
Total distance moved	three-way ANOVA	F (1, 72) = 2.580 P=0.1126	F (1, 72) = 3.225 P=0.0767	F (1, 72) = 1.386 P=0.2429	F (1, 72) = 0.2922 P=0.5905	F (1, 72) = 0.2801 P=0.5983	F (1, 72) = 0.0005583 P=0.9812	F (1, 72) = 1.387 P=0.2428

Supplementary Figure S6

	Type of test	F (DFn. DFd). P-value for Sex factor	F (DFn. DFd). P-value for MCC950 factor	F (DFn. DFd). P-value for METH factor	F (DFn. DFd). P-value for Sex x MCC950 factor	F (DFn. DFd). P-value for Sex x METH factor	F (DFn. DFd). P-value for MCC950 x METH factor	F (DFn. DFd). P-value for Sex x MCC950 x METH factor
Discrimination ratio	three-way ANOVA	F (1, 72) = 9.859 **P=0.0025**	F (1, 72) = 0.3723 P=0.5437	F (1, 72) = 0.003105 P=0.9557	F (1, 72) = 0.6494 P=0.4230	F (1, 72) = 0.3187 P=0.5742	F (1, 72) = 0.2771 P=0.6003	F (1, 72) = 0.03376 P=0.8547
